# Rhizosphere microbial patterns and climatic correlates of phenotypic variation in *Rosa roxburghii*

**DOI:** 10.3389/fmicb.2026.1762588

**Published:** 2026-03-31

**Authors:** Hongling Qin, Huan Gong, Xun Wang, Yu Liu, Wanling Song, Zhengbin Li, Heng Xiang

**Affiliations:** Guiyang Institute of Humanities and Technology, Guiyang, China

**Keywords:** climate factors, marker microbes, phenotypic variation, rhizosphere microbiome, *Rosa roxburghii*

## Abstract

Plant phenotypic variation represents an important expression of diversity among populations and their responses to heterogeneous environments. However, the factors associated with such variation remain incompletely characterized. In this study, we examined eight phenotypic traits of Rosa roxburghii across environmentally heterogeneous sites and characterized rhizosphere bacterial and fungal communities using 16S rRNA and ITS high-throughput sequencing. Marker taxa were identified, and statistical analyses were applied to explore associations between microbial patterns, climatic context, and phenotypic traits. Our results revealed significant differences among populations in fruit length, fruit width, and stem diameter. Microbial community analyses indicated inter-population divergence in both bacterial and fungal communities, with fungi showing relatively stronger compositional differentiation. Using complementary analytical approaches (LEfSe, LASSO, and sPLS-DA), we identified a consensus set of 12 marker taxa, including six bacterial and six fungal genera. Correlation analyses suggested that fungal markers exhibited broader associations with phenotypic traits than bacterial markers, and regression analyses indicated that fungal markers were statistically associated with variation in fruit size. Stem diameter showed associations with both climatic variables and microbial markers. Overall, phenotypic variation in R. roxburghii was associated with patterns in rhizosphere microbial communities and climatic context, highlighting complex relationships that warrant further investigation. This study contributes descriptive insights into plant–microbe and environment–phenotype associations and provides a foundation for future work aimed at elucidating underlying mechanisms.

## 1 Introduction

*Rosa roxburghii*, a perennial woody species native to southwestern China, produces fruits rich in vitamin C, polyphenols, and polysaccharides, conferring high nutritional and medicinal value and making it an important economic crop (Liu et al., 2016; Zhang et al., 2023; Jain et al., 2024). Wild populations of *R. roxburghii* harbor considerable genetic diversity and ecological adaptability, serving as valuable reservoirs of desirable traits (Lu et al., 2021; Liu et al., 2024a,b; He et al., 2025). However, current knowledge of phenotypic variation in *R. roxburghii* under natural conditions, as well as the environmental and biological drivers underlying such variation, remains limited. This knowledge gap constrains our understanding of the species’ adaptive strategies and ecological responses and hampers the effective utilization of wild germplasm resources in breeding programs.

Plant phenotypic variation represents an important strategy for coping with environmental heterogeneity and ecological pressures (Kou et al., 2014; Liu et al., 2018). Climatic factors, as major abiotic drivers, influence plant growth and development across spatial scales and can lead to phenotypic differentiation (Vizcaino-Palomar et al., 2020; Wang et al., 2022; Barker et al., 2025). Temperature, precipitation, and growing-season length directly affect physiological processes and morphological traits, shaping characteristics such as leaf morphology, stem diameter, flowering time, and fruit size (Limon et al., 2016; Liu et al., 2017). Along geographic gradients, climatic variation often results in pronounced phenotypic divergence among plant populations, a pattern widely recognized as a manifestation of local adaptation (Kardol et al., 2014; Bustamante et al., 2017; Pfennigwerth et al., 2017; Halbritter et al., 2018).

In addition to abiotic factors, increasing evidence suggests that belowground biotic components—particularly rhizosphere microbial communities—also play critical roles in shaping plant phenotypes and may modulate plant responses to environmental change (Yang et al., 2009; Bulgarelli et al., 2013; Fitzpatrick et al., 2018; Ortiz et al., 2020; Jacquiod et al., 2022). Rhizosphere microbes can enhance nutrient acquisition through nitrogen fixation, phosphorus solubilization, and the promotion of iron uptake (Fibach-Paldi et al., 2012; Finkel et al., 2017). They may also produce phytohormones such as indole-3-acetic acid (IAA), cytokinins, and gibberellins, or regulate ethylene levels via 1-aminocyclopropane-1-carboxylate (ACC) deaminase activity, thereby influencing root architecture, aboveground growth, flowering time, and biomass allocation (Fibach-Paldi et al., 2012). Furthermore, many rhizosphere microbes enhance plant tolerance to abiotic stresses (e.g., drought, salinity, and nutrient limitation) and increase resistance to biotic stresses, including pathogens and herbivores (Yang et al., 2009; Bittencourt et al., 2023). These functional roles suggest that rhizosphere microbes may contribute substantially to trait variation across environmental contexts and act as important biotic drivers of phenotypic differentiation.

Nevertheless, the relative roles of rhizosphere microbial communities and climatic factors in shaping phenotypic variation in wild *R. roxburghii* remain poorly understood. In this study, we investigated 25 wild *R. roxburghii* individuals from five environmentally heterogeneous sites. Key morphological traits were measured for each individual, and high-throughput sequencing was conducted to characterize their rhizosphere bacterial and fungal communities. These data were integrated with site-specific climatic information for comprehensive analysis. The objectives of this study were to: (1) determine whether wild *R. roxburghii* exhibits significant phenotypic variation across heterogeneous environments; and (2) identify marker rhizosphere microbes associated with environmental heterogeneity and quantify their relative contributions, together with climatic factors, to phenotypic variation. By integrating plant traits, microbial communities, and environmental variables, this study provides new insights into the biotic and abiotic drivers of phenotypic differentiation in wild *R. roxburghii*, offering a scientific basis for future breeding and conservation efforts.

## 2 Methods

### 2.1 Phenotypic measurement and variation analysis of *Rosa roxburghii* populations

Between July 6 and 9, 2025, five sampling sites were selected across Guizhou Province for the collection of wild *Rosa roxburghii* individuals. The topography of Guizhou Province generally exhibits a west-high, east-low distribution, and the five selected sites collectively cover the altitudinal gradient of *R. roxburghii* habitats. Moreover, the sites are evenly distributed across the east–west, north–south, and central regions of the province, thereby providing a representative reflection of environmental heterogeneity experienced by the species (Supplementary Figure S1). At each site, five healthy individuals at the fruit maturation stage were selected. Geographic coordinates were recorded, and phenotypic traits were subsequently measured (Supplementary Table S1). A total of eight traits were assessed in this study, with definitions and measurement protocols as follows:

Plant height was defined as the vertical distance from the soil surface to the highest point of the plant in its natural, fully extended state.

Crown width was measured as the average of the east–west and north–south diameters of the canopy.

Given that *R. roxburghii* is a shrub and breast height is difficult to measure, stem diameter was measured at 5–10 cm above the soil surface, avoiding the basal root-swelling region.

For each individual, five healthy, fully expanded mature leaves were collected to measure leaf length and leaf width, and five fruits were collected to measure fruit length, fruit width, and fresh weight. Mean values per individual were used for subsequent analyses, with final phenotypic data provided in Supplementary Table S2.

Kruskal-Wallis (KW) tests were conducted to identify traits exhibiting significant variation among populations across heterogeneous environments; these traits were prioritized in subsequent analyses of climate and microbial associations. Additionally, Wilcoxon tests were performed to assess pairwise phenotypic differences between populations.

### 2.2 Climate data collection and processing

Monthly historical climate data from January 2010 to December 2024 were obtained from the WorldClim database (Fick and Hijmans, 2017). Data corresponding to the geographic coordinates of each sampling site were extracted to represent the long-term climatic conditions experienced by the *Rosa roxburghii* populations at those locations. The climate dataset included temperature and precipitation variables, encompassing a total of 19 bioclimatic factors (Supplementary Tables S3, S4), with a spatial resolution of 2.5 arc-minutes.

### 2.3 DNA extraction and library preparation of *Rosa roxburghii* rhizosphere microbiota

Rhizosphere soil samples were collected from the previously phenotyped *Rosa roxburghii* individuals (one rhizosphere soil sample per individual; five individuals per site across five sites). First, debris and litter adhering to the roots were removed, and the roots were gently excavated. Loose soil was shaken off, and soil tightly adhering to the root surface (rhizosphere soil) was collected into sterile tubes. Samples were transported to the laboratory under a cold chain (0–4 °C) using ice packs. Upon arrival, samples were immediately aliquoted and stored at −80 °C until DNA extraction.

Genomic DNA was extracted from rhizosphere soil using the TGuideS96 magnetic bead-based soil DNA extraction kit. DNA concentrations were measured using a microplate reader, and the integrity of PCR-amplifiable DNA was evaluated by electrophoresis on 1.8% agarose gels.

For microbial community analysis, PCR amplification was performed targeting the bacterial 16S rRNA gene V3–V4 region using primers 341F (ACTCCTACGGGAGGCAGCA) and 806R (GGACTACHVGGGTWTCTAAT), and the fungal ITS1 region using the primer pair ITS1F (CTTGGTCATTTAGAGGAAGTAA) and ITS2 (GCTGCGTTCTTCATCGATGC), which amplifies the ITS1 region. PCR products were checked on 1.8% agarose gels, purified, and subsequently sequenced on the Illumina NovaSeq 6,000 platform using paired-end reads.

### 2.4 Sequencing data quality control and ASV construction

Sequencing data were processed using the DADA2 16S/ITS pipeline (Callahan et al., 2016). The workflow included quality control of raw reads, denoising, and construction of amplicon sequence variants (ASVs). Taxonomic annotation was performed using the SILVA database for bacteria and the UNITE database for fungi. Subsequent analyses of rhizosphere microbial communities across heterogeneous *Rosa roxburghii* populations were conducted in the R environment.

### 2.5 Diversity and compositional differences of rhizosphere microbial communities in *Rosa roxburghii* populations

First, ASV data were rarefied and zero-abundance taxa were removed. Alpha diversity analyses were then performed on the cleaned dataset using the MicrobiotaProcess R package to evaluate differences in rhizosphere microbial diversity across heterogeneous *R. roxburghii* populations (Xu et al., 2023). *p*-values were not adjusted for multiple comparisons. Microbial abundance data were subsequently converted to relative abundances, and the top ten phyla were extracted to generate a heatmap of community composition, allowing exploration of the distribution patterns of dominant phyla across populations in heterogeneous environments.

To preliminarily assess differences in rhizosphere microbial community composition among populations, we conducted a series of multivariate analyses based on Bray–Curtis dissimilarity matrices calculated from Hellinger-transformed community data. Permutational multivariate analysis of variance (PERMANOVA) was used to test whether community centroids differed significantly among populations. Because PERMANOVA can be sensitive to differences in within-group dispersion, we additionally performed permutational analysis of multivariate dispersions (*β*-dispersion) to evaluate the homogeneity of group variances. To further quantify the degree of separation between populations, we conducted analysis of similarities (ANOSIM), which assesses whether between-group dissimilarities exceed within-group dissimilarities. Given that each population corresponded to a geographically distinct site, we also performed partial Mantel tests to evaluate the relationship between community dissimilarity and geographic distance while controlling for elevation. All permutation-based analyses were conducted using 9,999 permutations.

To further assess compositional differences from multiple perspectives, four ordination methods—PCA (Principal Component Analysis), PCoA (Principal Coordinates Analysis), DCA (Detrended Correspondence Analysis), and CCA (Canonical Correspondence Analysis)—were applied. Additionally, hierarchical clustering based on Bray–Curtis distances (using the UPGMA method) was performed to evaluate the similarity of rhizosphere microbial composition among individual *R. roxburghii* plants.

### 2.6 Identification of marker microbes in rhizosphere communities of *Rosa roxburghii* across heterogeneous habitats using multiple supervised methods

At each taxonomic level, only taxa present at a relative abundance greater than 0.001 in at least 20% of samples (n ≥ 5) were retained to remove rare or low-abundance microbes, thereby reducing noise and improving the robustness of marker identification. Three complementary supervised statistical methods—LEfSe, LASSO regression, and sparse partial least squares discriminant analysis (sPLS-DA)—were then applied to the rhizosphere microbial communities to identify key taxa capable of discriminating among groups. In this study, taxa with LDA scores > 3 in LEfSe analyses were considered candidate marker taxa set 1 (Segata et al., 2011). Taxa with nonzero coefficients in LASSO regression were included in candidate marker taxa set 2 (Tibshirani, 2011), and taxa loading on the first principal component in sPLS-DA were considered candidate marker taxa set 3 (Cao et al., 2011). Considering that individual statistical methods may be subject to model assumptions or result instability, the final robust marker taxa were defined as the intersection of these three candidate sets.

### 2.7 Integrated analysis of climatic factors and key rhizosphere microbes on phenotypic variation

To avoid the confounding effects of collinearity among climatic factors, highly correlated variables (*r* > 0.7) within each climate category were excluded. Ultimately, four climatic factors were retained for subsequent analyses: two temperature-related factors (bio1 and bio2) and two precipitation-related factors (bio12 and bio13).

Spearman correlation analyses were first conducted to assess the relationships between climatic factors, bacterial markers, fungal markers, and phenotypic traits exhibiting significant differences. *p*-values were adjusted using the Benjamini–Hochberg false discovery rate (FDR) correction to control for multiple testing. Subsequently, multiple linear regression analyses were performed to identify key drivers of phenotypic variation. Variance partitioning analysis (VPA) was further applied to quantify the independent contributions of climatic factors, bacterial markers, and fungal markers to stem diameter variation, as well as their potential interactive effects.

## 3 Results

### 3.1 Phenotypic variation of *Rosa roxburghii* populations across heterogeneous habitats

**Figure 1:**
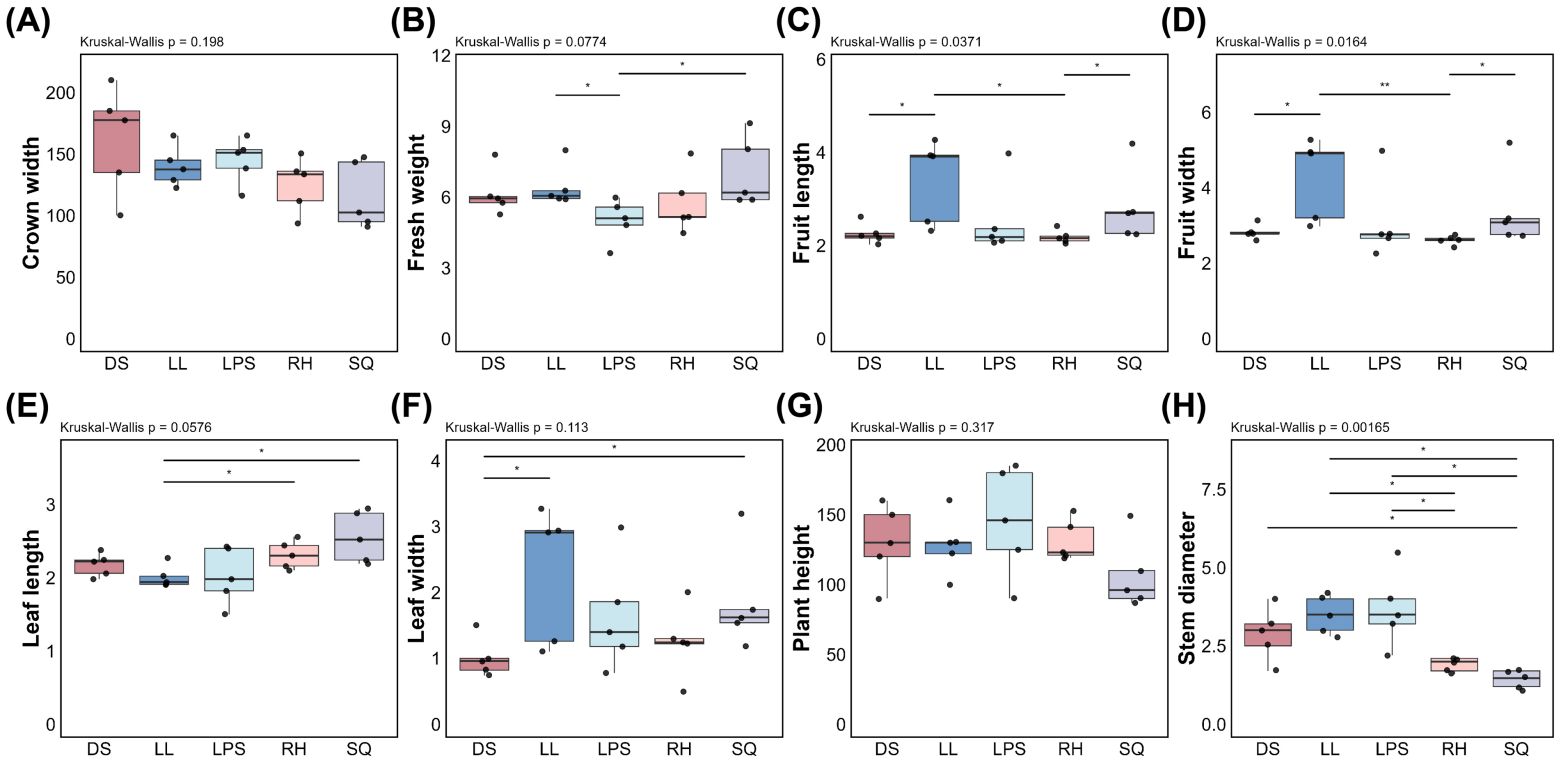
Phenotypic variation among different *Rosa roxburghii* populations. The *x*-axis and color scheme represent different *Rosa roxburghii* populations, while the *y*-axis indicates the measured values of each phenotypic trait. Black dots represent individual *Rosa roxburghii* plants. Kruskal-Wallis tests were performed for each trait, with *p* < 0.05 indicating significant differences among populations. Black horizontal bars indicate pairwise Wilcoxon test results. Significance levels are denoted as follows: “*” for *p* < 0.05 “**” for *p* < 0.01. and “***” for *p* < 0.001. No bar or asterisk is shown when the result is not significant.

We assessed eight phenotypic traits of *R. roxburghii* populations using the Kruskal–Wallis test. Among these traits, only fruit length, fruit width, and stem diameter showed significant differences among populations (Figures 1C,D,H). Fruit length and fruit width were significantly different (*p* = 0.0371 and *p* = 0.0164, respectively), and subsequent pairwise Wilcoxon tests revealed consistent patterns, with significant differences observed between DS and LL, as well as between LL and LPS, RH, and SQ. Bar plot comparisons indicated that the LL population exhibited greater fruit length and fruit width relative to most other populations. Stem diameter showed highly significant variation among populations (*p* = 0.00165), with most pairwise comparisons reaching statistical significance. In particular, the LL and LPS populations displayed significantly larger stem diameters than the remaining groups. Although fresh weight, leaf length, and leaf width did not exhibit overall significant differences among populations (*p* = 0.0774, *p* = 0.0576, and *p* = 0.113, respectively), variation in these traits was still evident at the population level. For instance, the LL population showed comparable leaf length but relatively greater leaf width compared to other groups (Figures 1B,E,F). No significant differences were detected among populations for crown width or plant height (Figures 1A,G).

Overall, the LL population tended to exhibit larger fruit size, greater leaf width, and increased stem diameter relative to other populations. However, this pattern was not consistently reflected in fresh weight, as the SQ population displayed the highest mean fresh weight.

### 3.2 Microbial diversity and compositional differences among *Rosa roxburghii* populations

**Figure 2:**
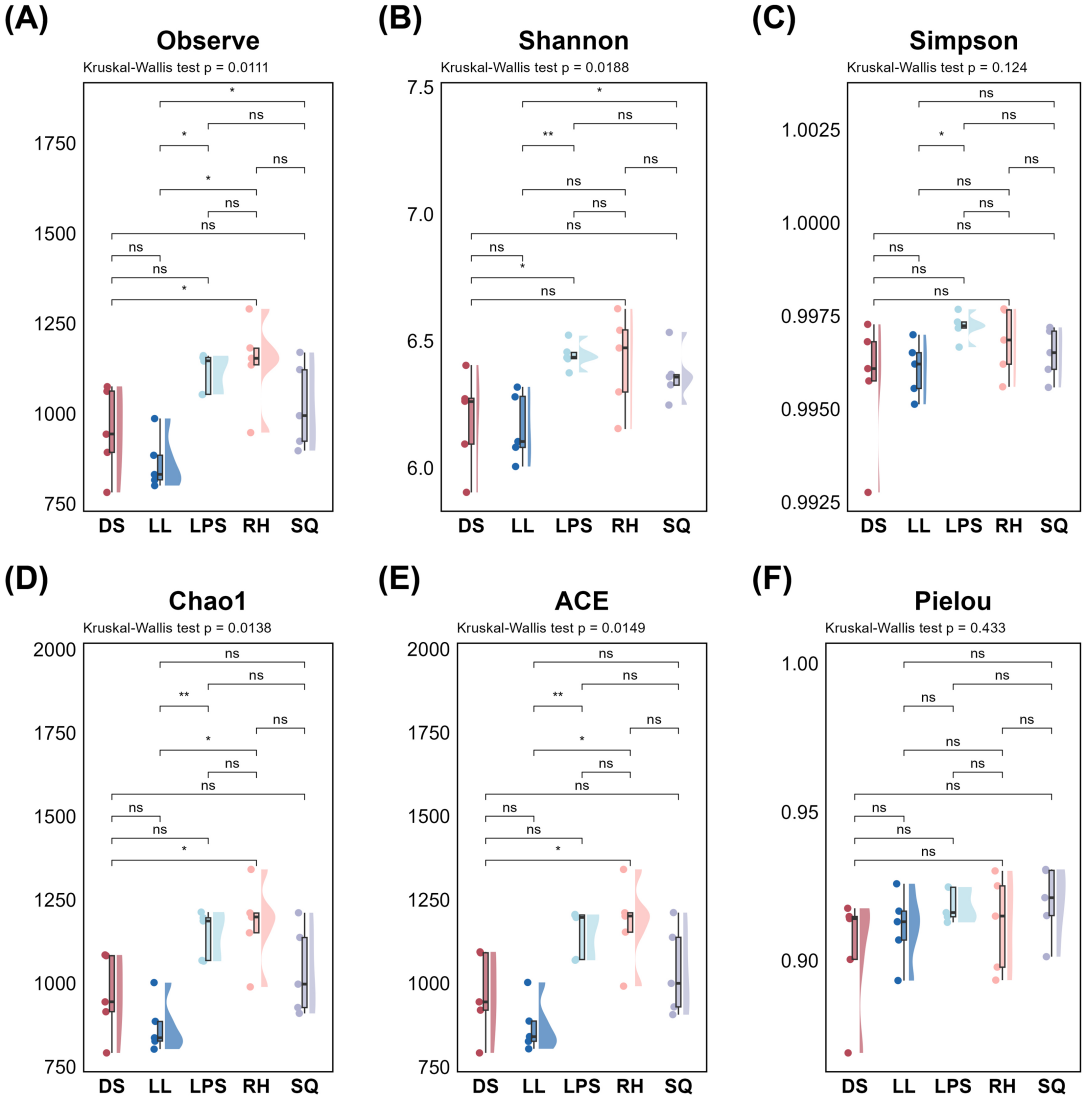
Alpha diversity of rhizosphere bacteria in *Rosa roxburghii* populations across heterogeneous habitats and results of pairwise comparisons. The *x*-axis and color represent different *Rosa roxburghii* populations, and the *y*-axis shows the diversity index values. Each circle represents an individual plant. Kruskal-Wallis tests were performed for all diversity indices, with *p* < 0.05, indicating significant differences among populations. Black horizontal bars indicate pairwise Wilcoxon test results. Significance levels are denoted as: “*” for *p* < 0.05, “**” for *p* < 0.01 and “***” for *p* < 0.001. Non-significant results are labeled as “ns.”

**Figure 3:**
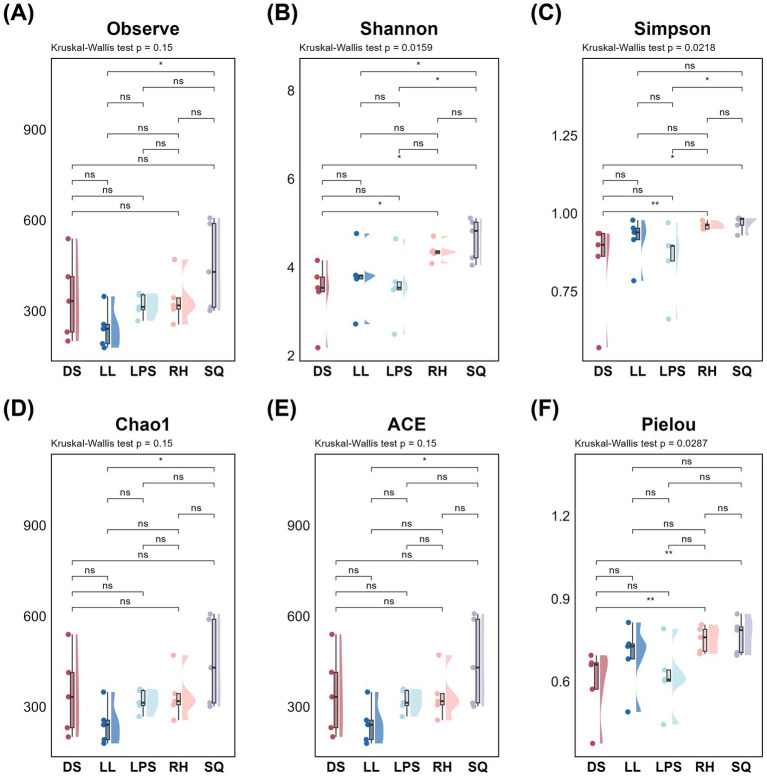
Alpha diversity of rhizosphere fungi in *Rosa roxburghii* populations across heterogeneous habitats and results of pairwise comparisons. The *x*-axis and color represent different *Rosa roxburghii* populations, and the *y*-axis shows the diversity index values. Each circle represents an individual plant. Kruskal-Wallis tests were performed for all diversity indices, with *p* < 0.05 indicating significant differences among populations. Black horizontal bars indicate pairwise Wilcoxon test results. Significance levels are denoted as: “*” for *p* < 0.05, “**” for *p* < 0.01, and “***” for *p* < 0.001. Non-significant results are labeled as “ns.”

Based on 16S rRNA gene and ITS sequencing, we characterized the rhizosphere microbial communities of *R. roxburghii* populations across heterogeneous environments. We first evaluated the alpha diversity of the rhizosphere microbiota. For bacteria, significant differences among populations were detected across all four alpha-diversity indices—Observed species, Shannon, Chao1, and ACE (K-W test, *p* < 0.05; unadjusted) (Figures 2A,B,D,E). The RH population exhibited the highest bacterial diversity (Observed species: 1153 ± 46; ACE: 1200.87 ± 57.91; Chao1: 1198.01 ± 59.08; Shannon: 6.47 ± 0.24), followed by LPS and SQ, whereas the DS and LL populations showed comparatively lower diversity. For fungi, significant inter-population differences were observed in the Shannon, Simpson, and Pielou indices (K-W test, p < 0.05; unadjusted) (Figures 3B,C,F). The SQ population exhibited the highest fungal diversity (Shannon: 4.82 ± 0.80; Simpson: 0.98 ± 0.02; Pielou: 0.79 ± 0.09). The RH and LL populations showed comparable Simpson and Pielou values to SQ, whereas LPS and DS displayed relatively lower fungal diversity. Overall, the RH population maintained relatively high alpha diversity for both bacteria and fungi, while the DS population consistently exhibited lower diversity across both microbial groups. The LPS population was notable for higher bacterial diversity, whereas SQ and LL tended to show comparatively greater fungal diversity.

**Figure 4:**
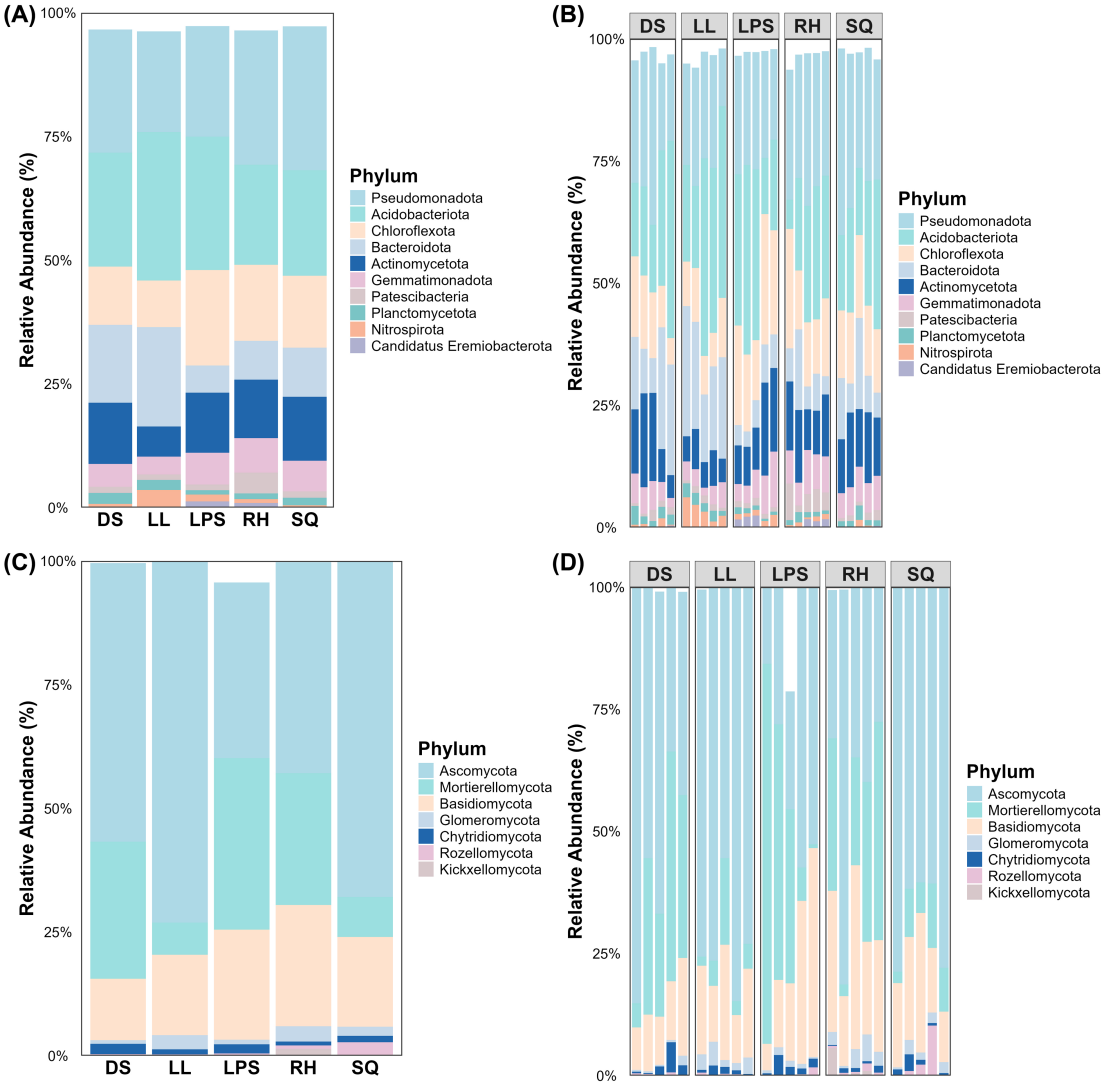
Top 10 phylum-level rhizosphere microbial compositions in *Rosa roxburghii* populations. Panels **(A,B)** show the average and individual-sample compositions of bacterial phyla, respectively, while panels **(C,D)** show the corresponding fungal compositions. The *x*-axis represents populations or individual samples, the y-axis indicates relative abundance, and colors denote different bacterial or fungal phyla.

We further quantified and visualized the ten most dominant phyla in the rhizosphere microbial communities (Figure 4). Among bacteria, the three most abundant phyla were Pseudomonadota, Acidobacteriota, and Chloroflexota, collectively accounting for more than 50% of total relative abundance in nearly all samples (Figures 4A,B). Although other bacterial phyla were present at lower relative abundances, their distribution patterns were relatively consistent across populations. The fungal community comprised seven phyla, with Ascomycota, Mortierellomycota, and Basidiomycota being dominant. Unlike the relatively even distribution observed in bacterial communities, Ascomycota and Mortierellomycota together accounted for ≥75% of total relative abundance in most populations. Notably, Ascomycota exhibited particularly high dominance in the LL and SQ populations (Figures 4C,D).

To further assess whether rhizosphere microbial composition differed significantly among populations, we performed PERMANOVA, ANOSIM, tests of multivariate homogeneity of dispersion (*β*-dispersion), and Mantel analyses (Table 1). PERMANOVA based on Bray–Curtis dissimilarity revealed significant differences in community composition among populations for both bacteria and fungi. Population grouping explained a moderate proportion of the total variance, accounting for 27.5% of variation in bacterial communities (*R*^2^ = 0.275, *p* < 0.001) and 24.4% in fungal communities (*R*^2^ = 0.244, *p* < 0.001). Tests of multivariate homogeneity of dispersion indicated no significant differences in within-group dispersion for either bacteria (*F* = 0.018, *p* = 0.999) or fungi (*F* = 0.192, *p* = 0.943), suggesting that the observed PERMANOVA results were not attributable to heterogeneity in dispersion. ANOSIM results were consistent with PERMANOVA, confirming significant separation among populations for both microbial groups. However, the magnitude of separation differed between bacteria and fungi. The ANOSIM R value for bacteria was 0.370 (*p* < 0.001), indicating moderate differentiation among populations, whereas fungi exhibited a higher R value of 0.565 (*p* < 0.001), reflecting stronger inter-population differentiation. Partial Mantel tests further demonstrated that community dissimilarity was significantly correlated with geographic distance after controlling for elevation in both bacterial (*r* = 0.351, *p* < 0.001) and fungal communities (*r* = 0.228, *p* = 0.0014). These results support the presence of a significant distance–decay relationship in rhizosphere microbial composition.

**Table 1:** Multivariate analyses (PERMANOVA, ANOSIM, *β*-dispersion, and Mantel tests) assessing differences in rhizosphere bacterial and fungal community composition among *Rosa roxburghii* populations.

Community	PERMANOVA (R^2^, P)	ANOSIM (R, P)	*β*-dispersion (F, P)	Mantel tests (R, P)
Bacteria	0.275, 0.0001	0.370, 0.0001	0.018, 0.999	0.351, 0.0001
Fungi	0.244, 0.0001	0.565, 0.0001	0.192, 0.943	0.228, 0.0014

Community refers to the type of microbial community analyzed (bacterial or fungal). PERMANOVA and ANOSIM were conducted based on Bray–Curtis dissimilarity with 9,999 permutations. Homogeneity of multivariate dispersion was tested using permutational analysis of multivariate dispersions (9,999 permutations). Mantel tests represent partial Mantel correlations between community dissimilarity and geographic distance while controlling for elevation.

**Figure 5:**
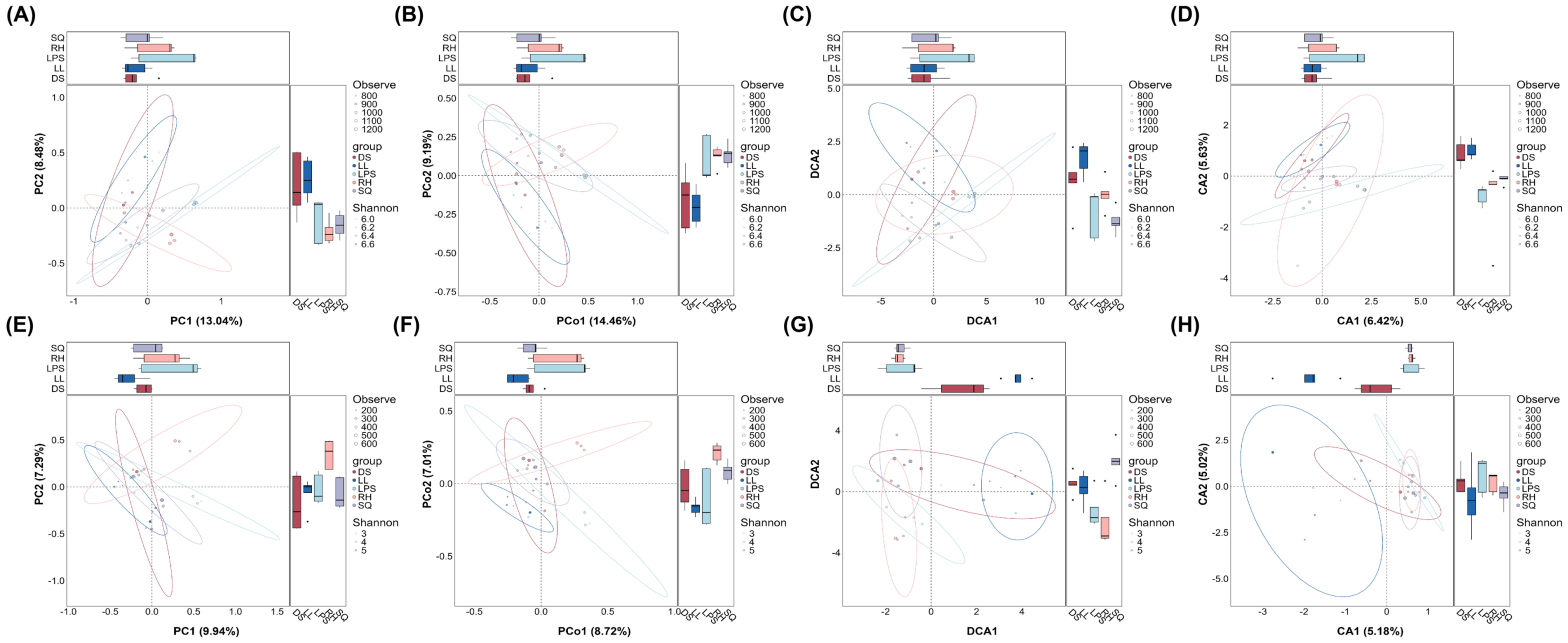
Differences in rhizosphere microbial community composition among *Rosa roxburghii* populations across heterogeneous habitats. Panels **(A–D)** show bacterial community ordinations using PCA, PCoA, DCA, and CCA, respectively, while panels **(E–H)** show fungal community ordinations. The *x*- and *y*-axes represent the main ordination axes and their explained variance. Colors indicate different *Rosa roxburghii* populations. Each point represents an individual plant, with point size and transparency corresponding to the Observe and Shannon diversity indices, respectively, to convey alpha diversity information.

**Figure 6:**
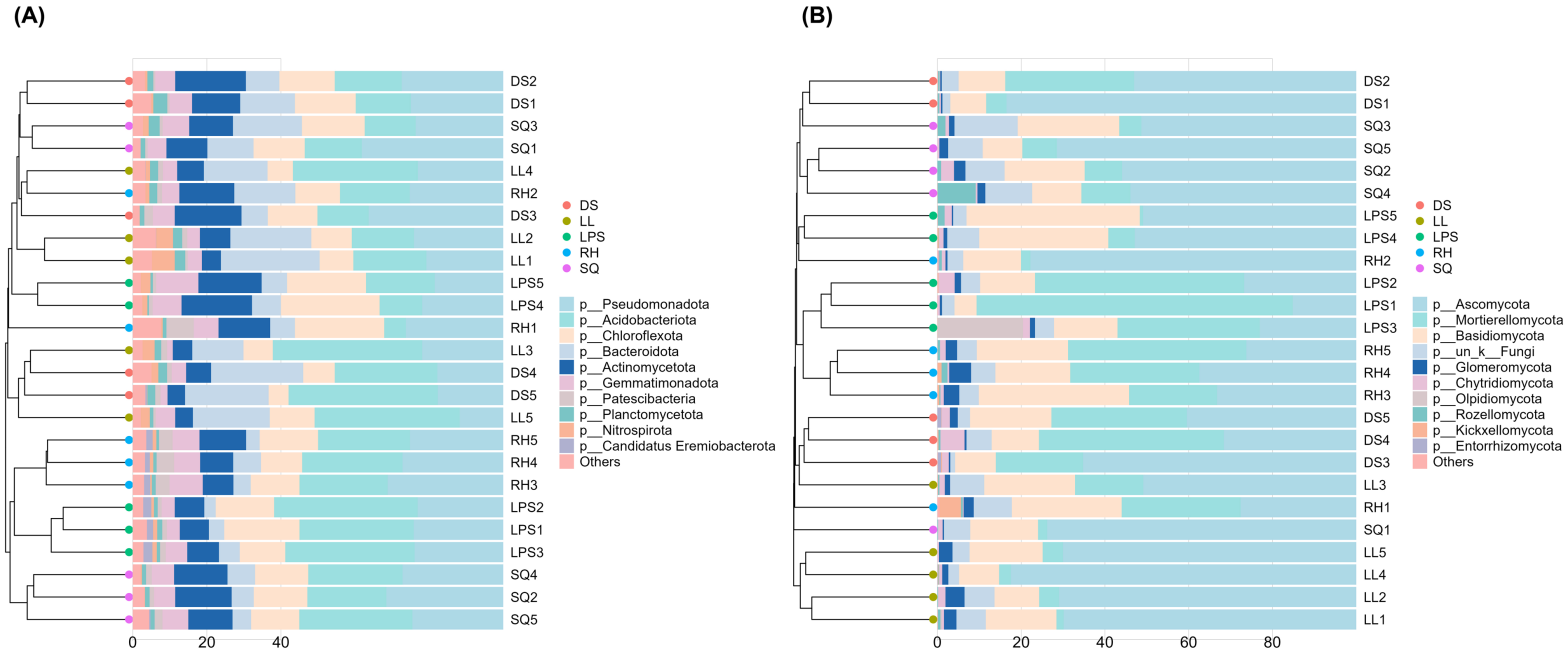
Hierarchical clustering of *Rosa roxburghii* rhizosphere microbial communities based on Bray-Curtis distances of phylum-level abundances (**A**, bacteria; **B**, fungi). The *x*-axis represents relative abundance, and the *y*-axis indicates individual plants. Colored dots on the left dendrogram indicate population origin, with closer distances reflecting greater similarity in phylum-level composition between individuals. The stacked bar plots in the center depict the phylum-level composition of rhizosphere microbes for each individual.

We applied multiple ordination approaches—including PCA, PCoA, DCA, and CCA—to explore differences in rhizosphere microbial community composition among *R. roxburghii* populations (Figure 5). Bacterial community composition was generally difficult to distinguish among populations. In particular, the DS and RH groups consistently exhibited substantial overlap in ordination space with other populations (Figures 5A–D). Even after excluding these two populations, separation among the remaining groups remained weak, indicating limited compositional differentiation in bacterial communities. In contrast, fungal communities displayed pronounced structuring among populations. Both DCA and CCA ordinations revealed clear variation in rhizosphere fungal composition across populations (Figures 5G,H). In the DCA plot, the LL group was distinctly separated from most other populations, showing only limited overlap with DS, whereas overlap among other populations was less extensive than that observed in bacterial communities. CCA further supported the distinctiveness of the LL group, although the relative distances among some of the remaining groups were slightly reduced compared to the DCA results. Consistent with the ordination analyses, UPGMA clustering based on Bray–Curtis dissimilarities (average linkage) revealed contrasting patterns between microbial groups. Bacterial communities at the phylum level showed no clear clustering by population (Figure 6A), whereas fungal communities exhibited more evident population-level structure, with samples from the SQ and LL populations clustering closely together (Figure 6B).

### 3.3 Associations between core rhizosphere microbes, climatic variables, and phenotypic variation in *Rosa roxburghii* populations

**Figure 7:**
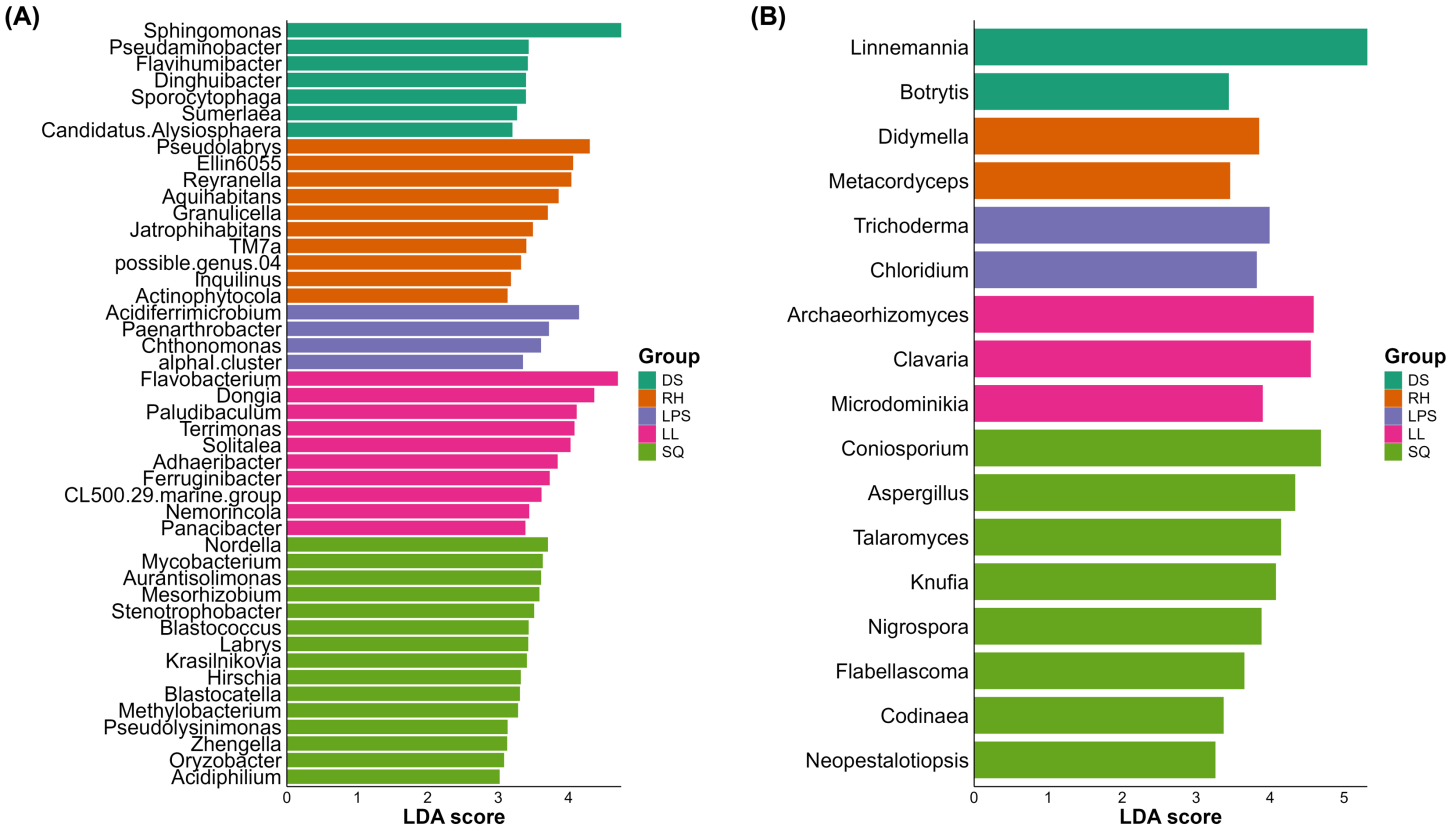
Genus-level key rhizosphere microbes identified by LEfSe (**A**, bacteria; **B**, fungi). The *x*-axis represents LDA scores, and the *y*-axis shows taxon names. Colors indicate the groups in which each taxon is significantly enriched.

**Figure 8:**
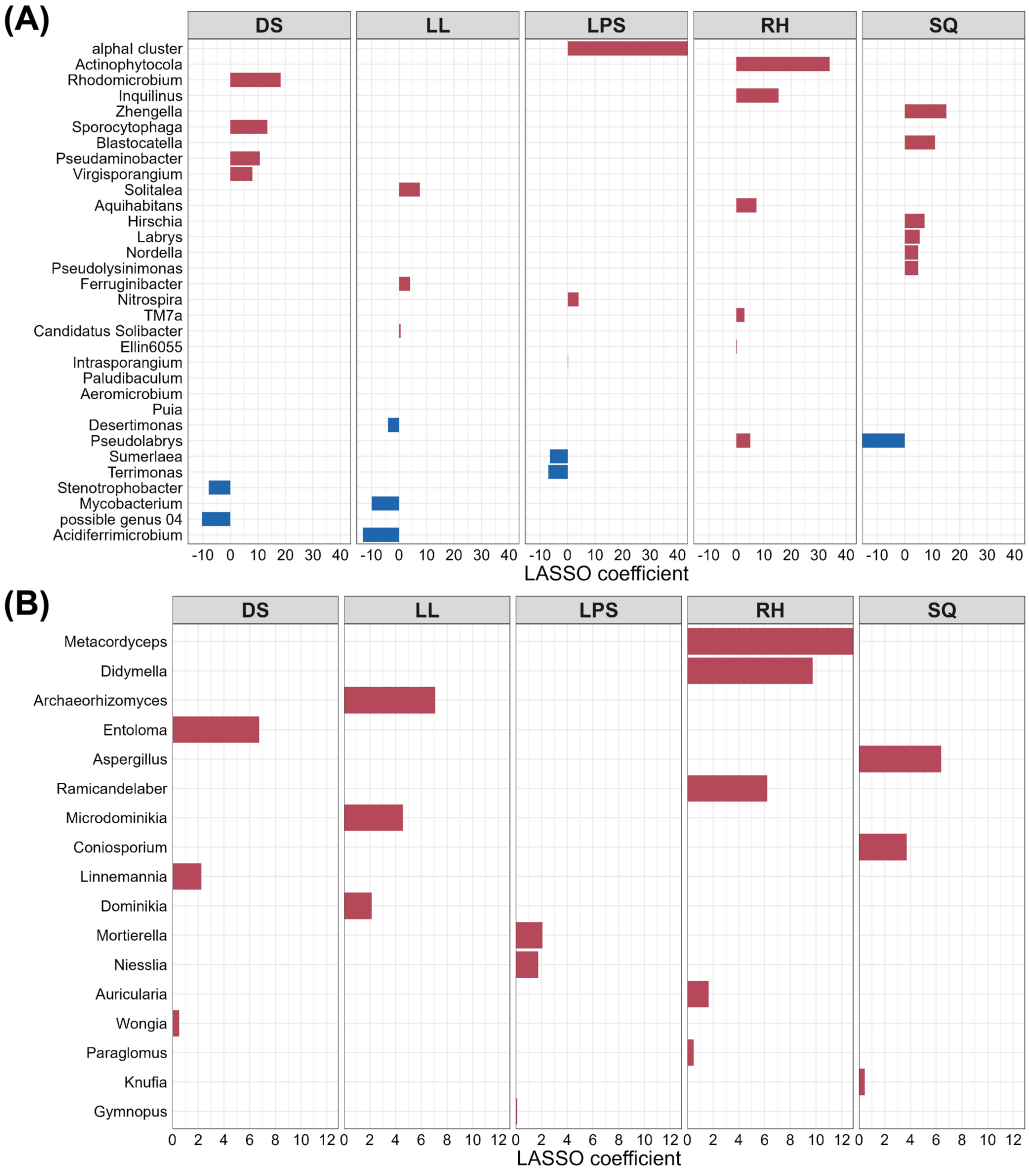
Key rhizosphere microbes selected by LASSO with nonzero λ coefficients (**A**, bacteria; **B**, fungi). The *x*-axis represents the λ coefficient values, and the *y*-axis shows taxon names. Colors indicate the direction of effect: red for positive and blue for negative.

**Figure 9:**
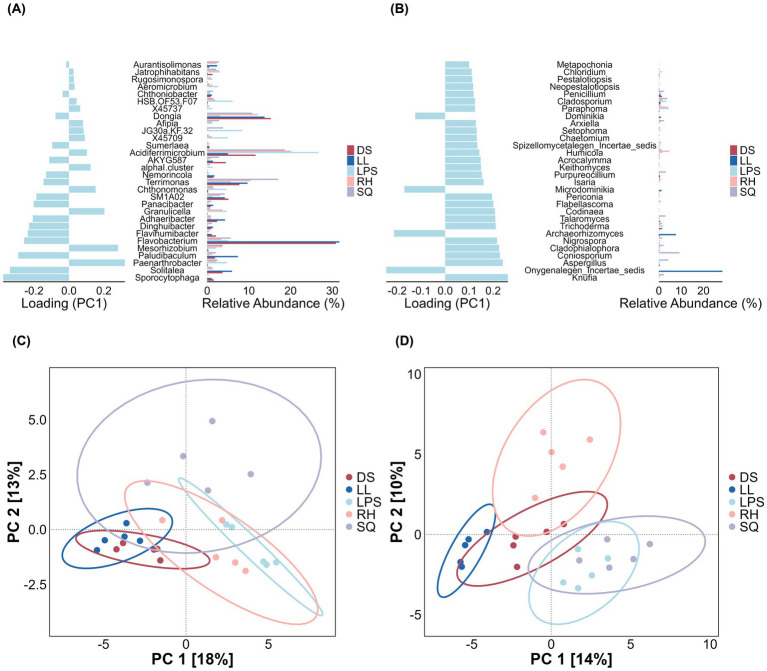
Key rhizosphere microbes identified by sPLS-DA. Panels **(A,B)** show the weights of key taxa on the first principal component for bacteria and fungi (Top 30), respectively, along with their relative abundances across groups. Panels **(C,D)** depict sample distributions based on bacterial and fungal sPLS-DA results, illustrating the discriminatory power of these key taxa among heterogeneous *Rosa roxburghii* populations.

**Figure 10:**
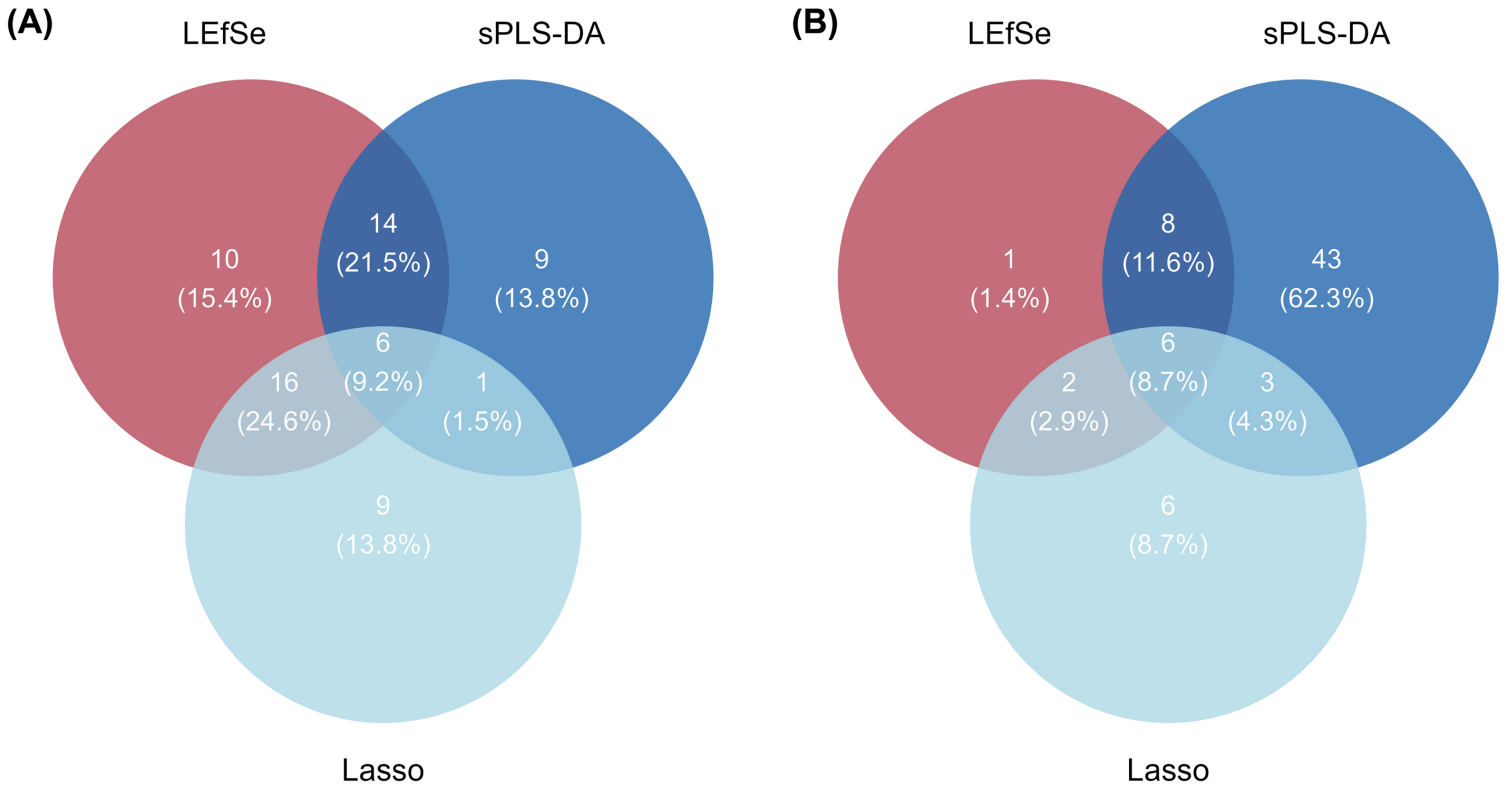
Intersection of key microbes identified by multiple methods. **(A)** Six bacterial marker genera were selected, and **(B)** six fungal marker genera were identified.

To identify core marker rhizosphere microbes that represent key inter-population differences, we applied three complementary supervised methods-LEfSe, LASSO regression, and sPLS-DA-at the genus level for both bacteria and fungi, and the intersection of their results was taken as the set of core differential taxa. LEfSe identified 46 bacterial genera and 17 fungal genera as candidate markers (Figures 7, 10). LASSO selected 32 bacterial and 17 fungal genera (Figures 8, 10), while sPLS-DA identified 30 bacterial and 60 fungal genera (Figures 9, 10).

By taking the intersection of the results from the three methods, we ultimately identified six bacterial genera and six fungal genera as core markers. Among the bacteria, three genera—*Solitalea*, *Sporocytophaga*, and *Terrimonas*—are classified within the phylum Bacteroidota, whereas the other three—*Acidiferrimicrobium*, *Paludibaculum*, and *Sumerlaea*—are affiliated with the phyla Actinomycetota, Acidobacteriota, and Sumerlaeota, respectively (Supplementary Figure S2). Notably, the three Bacteroidota genera exhibited relatively higher abundance in the DS and LL groups. For fungi, four marker genera—*Aspergillus*, *Coniosporium*, *Knufia*, and *Archaeorhizomyces*—belong to the phylum Ascomycota (Supplementary Figure S3). Except for *Archaeorhizomyces*, the Ascomycota genera were predominantly more abundant in the SQ group. In addition, *Microdominikia* (phylum Glomeromycota) and *Archaeorhizomyces* were almost exclusively enriched in the LL group.

**Figure 11:**
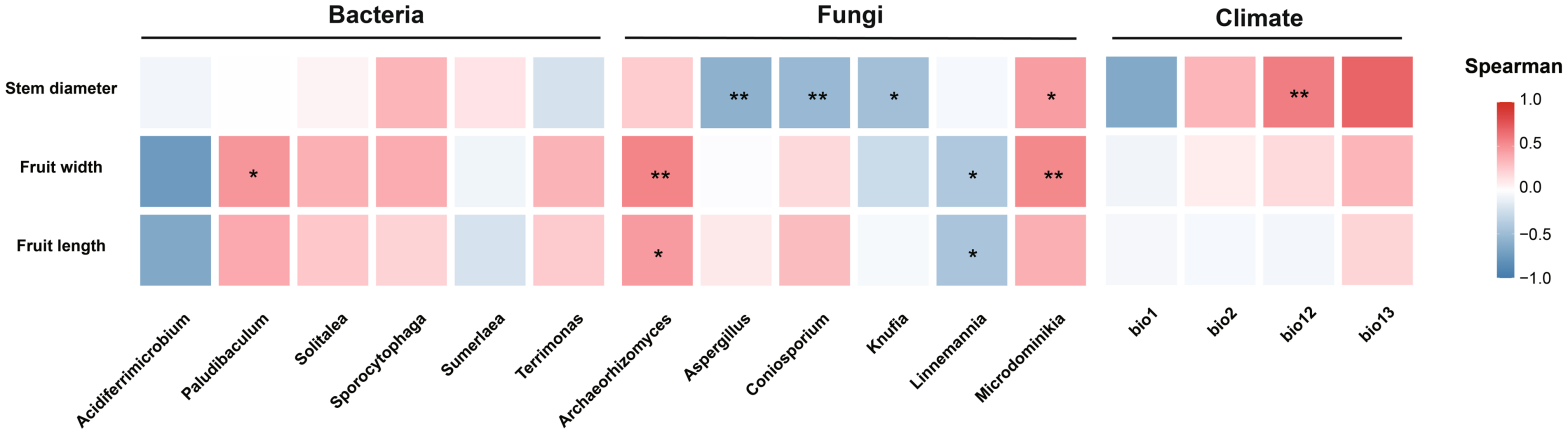
Spearman correlation analysis between differential phenotypic traits of *Rosa roxburghii* populations and marker rhizosphere microbes (bacteria and fungi) as well as climate factors. The *x*-axis represents microbial genera and climate variables, while the *y*-axis represents the phenotypic traits. Colors indicate the strength and direction of correlations. Significance levels are denoted as “*” for *p* < 0.05, “**” for *p* < 0.01, and “***” for *p* < 0.001. Non-significant correlations are left blank.

We assessed the associations between marker rhizosphere microbial taxa and climatic factors with the phenotypic differentiation observed among *R. roxburghii* populations. (Figure 11). Among bacterial markers, only *Paludibaculum* showed a significant positive correlation with fruit width, and among climatic factors, only Bio12 (annual precipitation) exhibited a significant positive association with stem diameter. In contrast to bacteria and climatic variables, fungal marker taxa displayed broader and more consistent relationships with plant phenotypes. Notably, both *Archaeorhizomyces* and *Linnemannia* were significantly associated with fruit width and fruit length; however, the direction of association differed—*Archaeorhizomyces* showed a positive correlation, whereas *Linnemannia* showed a negative one. *Microdominikia* exhibited significant positive correlations with both stem diameter and fruit width. The remaining fungal markers—*Aspergillus*, *Coniosporium*, and *Knufia*—were each significantly negatively correlated with stem diameter.

Further multiple linear regression analyses indicated that among all climatic variables, only the model for stem diameter reached statistical significance, explaining approximately 67.2–72.7% of its variation (*R*^2^ = 0.727, Adj. *R*^2^ = 0.672, *p* < 0.001). Within this model, bio1 was negatively associated with stem diameter (*β* = −1.942, *p* = 0.035), whereas bio12 was positively associated with stem diameter (*β* = 0.886, *p* = 0.039). The regression coefficients indicated opposite directions of association, with the magnitude of the coefficient for bio1 being slightly larger. In contrast, no statistically significant associations between climatic variables and fruit length or fruit width were detected.

Regarding marker rhizosphere microorganisms, regression models for fruit length, fruit width, and stem diameter were all statistically significant, with adjusted *R*^2^ values of 40.8, 44.4, and 58.6%, respectively. *Coniosporium* was the only microbial taxon that showed significant associations with all three phenotypic traits, displaying positive associations with fruit length and fruit width and a negative association with stem diameter. In addition, *Aspergillus* was negatively associated with fruit width. The bacterial genera *Solitalea* and *Sporocytophaga* were positively and negatively associated with stem diameter, respectively, while *Microdominikia* showed a significant positive association with stem diameter.

**Figure 12:**
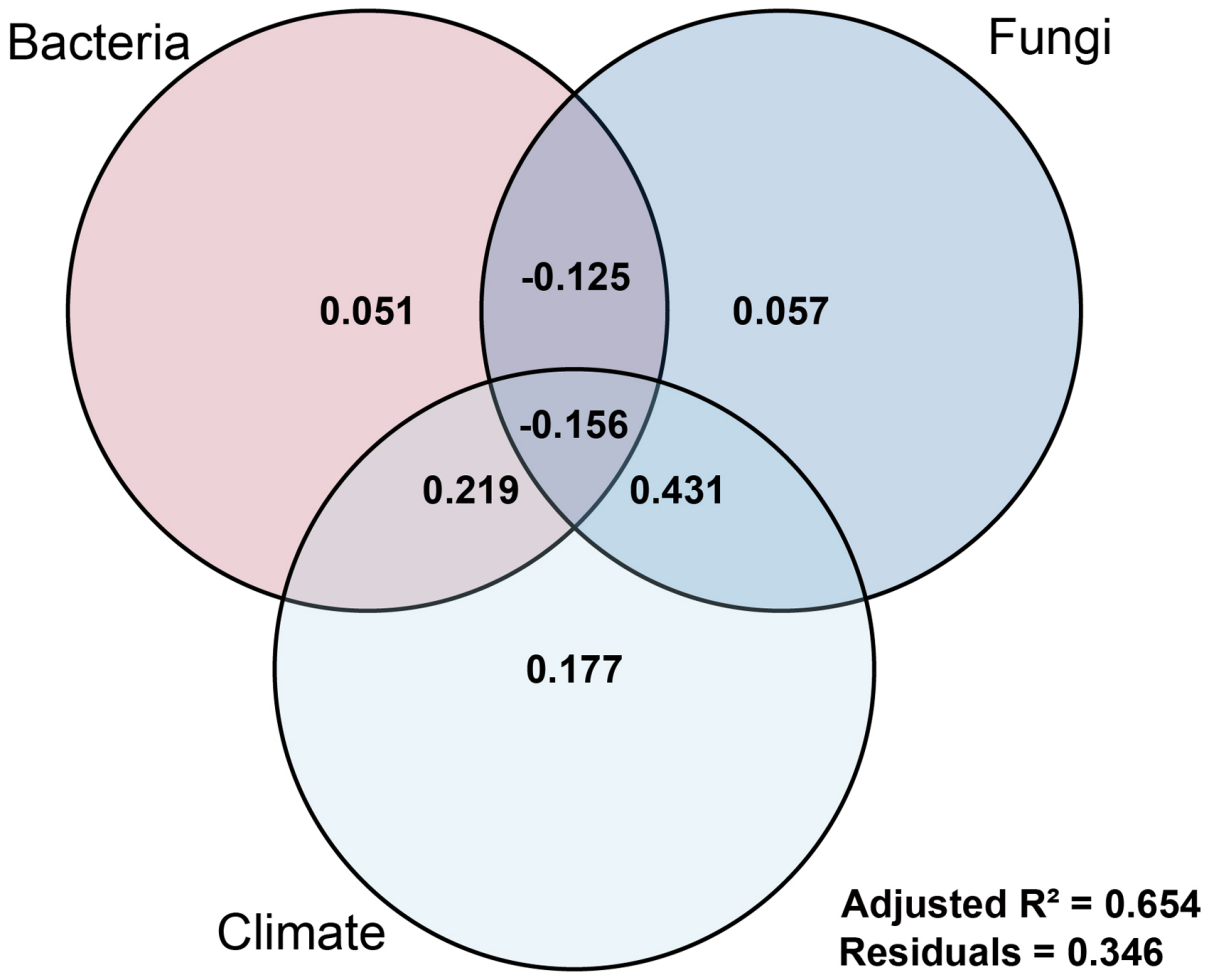
Variance partitioning analysis (VPA) showing the independent and interactive contributions of marker bacteria, marker fungi, and climatic factors to stem diameter variation in *Rosa roxburghii* populations. The VPA results show that the full model explains 65.4% of variation (adjusted *R*^2^ = 0.654). Climate exhibited the largest unique contribution (0.177), followed by fungi (0.057) and bacteria (0.051). Substantial shared components (e.g., climate-fungi = 0.431; climate-bacteria = 0.219) indicate interactive effects among drivers, while negative higher-order fractions reflect suppression effects inherent to variance partitioning. The residual (0.346) represents unexplained variation.

From a trait-level perspective, fruit length and fruit width were statistically associated with fungal variables, whereas stem diameter showed associations with bacterial variables, fungal variables, and climatic variables. Variance partitioning analysis (VPA) indicated that variation in stem diameter was jointly accounted for by these three sets of explanatory variables, with an adjusted *R*^2^ of 0.654, suggesting that the model explained 65.4% of the observed variation (Figure 12). Among the unique fractions, climatic variables accounted for a larger independent proportion (0.177) compared with fungi (0.057) and bacteria (0.051). Substantial shared fractions were also observed: the bacteria–fungi component was −0.125, the climate–fungi component was 0.431, and the climate–bacteria component was 0.219, whereas the higher-order shared fraction among all three variable sets was −0.156. These partitioning results indicate statistical overlap and collinearity among explanatory variables rather than independent effects. The sum of all unique and shared fractions equaled 0.654, consistent with the adjusted R^2^. The remaining unexplained proportion (0.346) may reflect additional unmeasured environmental variables, including site-level soil physicochemical properties or microclimatic conditions, as well as stochastic variation. Accordingly, these results should be interpreted as describing patterns of association rather than mechanistic attribution.

## 4 Discussion

### 4.1 Reproductive trait differentiation among *Rosa roxburghii* populations across heterogeneous environments

*R. roxburghii* populations exhibited significant phenotypic variation across heterogeneous environments, particularly in fruit length, fruit width, and stem diameter (Figures 1C,D,H). Notably, two of these traits are directly related to reproduction, suggesting that environmental heterogeneity may preferentially influence differentiation in reproductive allocation (Santos-del-Blanco et al., 2013).

Although plant height and crown width did not differ significantly among populations, the LL population displayed especially pronounced reproductive characteristics. First, fruit size (length and width) was greater than in most other populations (Figures 1C,D). Second, fruit fresh weight ranked among the highest (Figure 1B), although differences were not statistically significant. In addition, LL exhibited a relatively large stem diameter, which may reflect enhanced structural support and resource transport capacity (Tang et al., 2022), potentially facilitating the development of larger fruits. The SQ population also exhibited prominent reproductive traits comparable to those of LL, but with a contrasting growth pattern. Stem diameter was the smallest among all populations, whereas leaf area was exceptionally large, with both leaf length and width substantially greater than in other populations (Figures 1E,F). This pattern suggests that SQ may rely more heavily on expanded photosynthetic surfaces and assimilate production (Sun et al., 2023; Li et al., 2025) to sustain fruit development, representing a reproductive strategy distinct from that of LL.

Overall, environmental heterogeneity appears to promote divergence in reproductive traits among *R. roxburghii* populations, yet different populations seem to adopt alternative growth–reproduction allocation strategies to achieve similar reproductive outputs.

### 4.2 Stronger inter-population divergence in fungal than in bacterial communities among *Rosa roxburghii* populations

Although *R. roxburghii* populations from heterogeneous environments exhibited significant differences in rhizosphere microbial alpha diversity for both bacteria and fungi (Figures 2, 3), it is important to note that the dominant bacterial phyla were relatively evenly distributed, showing minimal differences among populations (Figures 4A,B). In contrast, the distribution of dominant fungal phyla was markedly heterogeneous. For instance, Ascomycota, which is highly abundant in the LL and SQ populations—previously noted for their superior reproductive traits—represented a particularly large proportion of the fungal community in these groups (Figures 4C,D). Conversely, the second dominant phylum, Mortierellomycota, was relatively less abundant in these two populations. This pattern suggests a potential influence of rhizosphere fungal distribution on phenotypic traits (Bolin, 2025).

Significant differences in rhizosphere microbial composition were observed among *R. roxburghii* populations (Table 1), indicating that microbial communities were structured by population-level heterogeneity. PERMANOVA results demonstrated that both bacterial and fungal communities exhibited significant inter-population variation, and these differences were not attributable to heterogeneity in within-group dispersion, as confirmed by non-significant *β*-dispersion tests. This suggests that the observed community differentiation reflects genuine shifts in community centroids rather than differences in group variance. The degree of community separation differed between microbial groups. The ANOSIM R value for bacteria (*R* = 0.370) indicated moderate differentiation among populations, whereas fungi exhibited a higher *R* value (*R* = 0.565), reflecting stronger inter-population separation. This pattern implies that fungal communities may be more sensitive to environmental or host-related factors, resulting in clearer compositional differentiation across populations, while bacterial communities displayed more moderate structuring and greater compositional overlap. Partial Mantel tests further revealed significant correlations between community dissimilarity and geographic distance after controlling for elevation for both bacteria (*r* = 0.351, *p* < 0.001) and fungi (*r* = 0.228, *p* = 0.0014). The stronger distance–decay relationship observed for bacteria suggests that spatial processes may exert a relatively greater influence on bacterial community assembly, potentially reflecting dispersal limitation or continuous spatial gradients in environmental conditions. In contrast, the weaker distance–decay pattern in fungi implies that additional factors—such as host selection or niche differentiation—may play a larger role in shaping fungal community structure.

These statistical results were highly consistent with multiple ordination analyses: fungal communities showed clearer clustering among populations, with much less overlap in confidence intervals compared to bacteria (Figures 5G,H). Furthermore, hierarchical clustering based on fungal Bray–Curtis distances supported this conclusion, as samples from certain populations (e.g., SQ and LL, n = 4) consistently clustered within the same branch (Figure 6B), indicating higher community consistency and internal similarity relative to bacteria (Figure 6A).

Collectively, these results indicate that rhizosphere fungal communities in *R. roxburghii* exhibited more pronounced population-level differentiation and were more responsive to heterogeneous environmental conditions. Bacterial communities also showed significant inter-population variation and spatial structuring, although the degree of differentiation was comparatively moderate and characterized by greater compositional overlap among populations. This may be attributed to the higher host specificity, stronger niche differentiation, and lower functional redundancy of fungi in plant interactions (Chen et al., 2022; Tang et al., 2022), whereas bacteria generally exhibit higher functional redundancy and broader ecological tolerance, resulting in weaker spatial structuring (Song et al., 2025).

Nonetheless, based on the current findings, fungi appear to have greater potential as sensitive indicator taxa for distinguishing among *Rosa roxburghii* populations or explaining environmental differences, whereas bacterial variation among populations may be more influenced by microenvironmental fluctuations, stochastic dispersal, or background soil noise (Wagg et al., 2019).

### 4.3 Core fungal genera exhibited broader associations with phenotypic variation in *Rosa roxburghii*

Using multiple unsupervised approaches, we identified marker taxa that were most responsive to environmental heterogeneity, including six bacterial genera and six fungal genera (Figures 10A,B). Subsequent correlation analyses among these marker taxa, climatic variables, and key phenotypic traits revealed that each fungal marker genus was significantly associated with at least one focal trait. In contrast, bacterial markers and climatic variables exhibited fewer significant associations (Figure 11). These results suggest that fungal taxa may be more broadly linked to phenotypic variation in *R. roxburghii*, although the correlational nature of the analysis precludes causal inference.

To examine the relative statistical associations of different variable sets while accounting for potential collinearity, multiple linear regression models were constructed. The regression analyses provided trait-specific patterns of association. Rhizosphere microbial variables were significantly associated with variation in all three key traits. Fungal variables accounted for a substantial proportion of the variation in fruit length and fruit width (adjusted *R*^2^ = 40.8 and 44.4%, respectively; Table 3), whereas bacterial variables were more strongly associated with stem diameter (adjusted R^2^ = 58.6%). Climatic variables exhibited a different pattern of association. They were not significantly associated with fruit size but showed significant associations with stem diameter, with bio1 (Annual Mean Temperature) and bio12 (Annual Precipitation) identified as significant predictors. The climatic model accounted for 67.2% of the variation in stem diameter, slightly exceeding the proportion explained by microbial variables alone (Table 2). Variance partitioning analysis (VPA) further quantified the distribution of explained variation among variable sets. In terms of independent fractions, climatic variables accounted for a larger unique proportion of the explained variation in stem diameter (Figure 12). However, substantial shared fractions between climatic and microbial variables were also observed. In particular, the climate–fungi shared component accounted for 0.431 of the explained variation (Adj. *R*^2^ = 0.654). These results indicate statistical overlap and covariation among climatic and microbial variables in relation to stem diameter. It is important to note, however, that VPA reflects the partitioning of variation among the variables included in the model and does not represent the complete set of ecological drivers influencing phenotypic traits. Additional factors not incorporated in the analysis—such as soil physicochemical properties, host genetic background, and other unmeasured environmental variables—may also contribute to phenotypic variation (Daws et al., 2020; Bahram et al., 2021). Therefore, the independent and shared fractions reported here should be interpreted as conditional on the variables analyzed rather than as comprehensive estimates of the full ecological determinants of trait variation.

**Table 2:** Stepwise multiple linear regression revealing the effects of climatic factors on phenotypic variation in *Rosa roxburghii* populations.

Trait	Factor	*β*	SE	*t*	*P*	*R* ^2^	Adj. *R*^2^	Module *P*
Fruit length	bio1	−0.901	1.598	−0.563	0.579	0.047	−0.143	0.907
	bio2	0.719	1.780	0.404	0.691			
	bio12	0.381	0.749	0.508	0.617			
	bio13	−1.148	2.592	−0.443	0.663			
Fruit width	bio1	−0.574	1.590	−0.361	0.722	0.057	−0.132	0.873
	bio2	0.280	1.771	0.158	0.876			
	bio12	0.310	0.745	0.416	0.682			
	bio13	−0.558	2.579	−0.216	0.831			
Stem diameter	bio1	−1.942	0.856	−2.267	0.035	0.727	0.672	<0.001
	bio2	1.593	0.954	1.670	0.110			
	bio12	0.886	0.401	2.208	0.039			
	bio13	−1.994	1.389	−1.436	0.166			

Trait refers to the phenotypic trait used to characterize differences among *Rosa roxburghii* populations. Factor indicates the climatic predictor included in the final regression model. *β* is the regression coefficient, with negative values showing negative relationships and positive values showing positive relationships. SE is the standard error of *β*. *t* is the *t*-statistic for testing significance, and *P* is the associated significance level. *R*^2^ represents the proportion of variance explained by the model, and Adj. *R*^2^ is the adjusted *R*^2^ accounting for model complexity. Model *P* indicates the overall significance of the regression model.

**Table 3:** Stepwise multiple linear regression revealing the effects of marker rhizosphere microbes (bacteria and fungi) on phenotypic variation in *Rosa roxburghii* populations.

Trait	Factor	*β*	SE	*t*	*P*	*R* ^2^	Adj. *R*^2^	Module *P*
Fruit length	*Acidiferrimicrobium^B^*	−7.876	4.473	−1.761	0.094	0.531	0.408	0.009
	*Sporocytophaga^B^*	1.904	1.351	1.410	0.175			
	*Aspergillus*	−2.491	1.260	−1.977	0.063			
	*Coniosporium*	2.568	1.001	2.565	0.019			
	*Linnemannia*	−2.078	1.520	−1.368	0.187			
Fruit width	*Acidiferrimicrobium^B^*	−7.705	4.335	−1.777	0.091	0.560	0.444	0.005
	*Sporocytophaga^B^*	2.203	1.309	1.683	0.109			
	*Aspergillus*	−2.720	1.221	−2.227	0.038			
	*Coniosporium*	2.623	0.970	2.704	0.014			
	*Linnemannia*	−2.065	1.473	−1.402	0.177			
Stem diameter	*Solitalea^B^*	−4.488	1.718	−2.613	0.018	0.707	0.586	0.001
	*Sporocytophaga^B^*	3.589	1.125	3.191	0.005			
	*Terrimonas^B^*	−3.577	2.356	−1.518	0.147			
	*Aspergillus*	−1.637	1.178	−1.390	0.183			
	*Coniosporium*	−2.252	0.867	−2.597	0.019			
	*Linnemannia*	−1.574	1.229	−1.281	0.217			
	*Microdominikia*	3.348	1.216	2.753	0.014			

Trait refers to the phenotypic trait used to characterize differences among *Rosa roxburghii* populations. Factor indicates the rhizosphere microbial predictors included in the final regression model. Variables marked with the superscript “*B*” denote bacteria, whereas all others represent fungi. *β* is the regression coefficient, with negative values showing negative relationships and positive values showing positive relationships. SE is the standard error of *β*. *t* is the *t*-statistic for testing significance, and P is the associated significance level. *R*^2^ represents the proportion of variance explained by the model, and Adj. *R*^2^ is the adjusted *R*^2^ accounting for model complexity. Model *P* indicates the overall significance of the regression model.

### 4.4 Limitations and future directions

This study integrates phenotypic assessment, rhizosphere microbial profiling, and climatic variables to explore environment–microbiome–phenotype associations in wild *R. roxburghii* populations. While the results reveal clear patterns of population differentiation and statistically robust associations among microbial communities, environmental factors, and plant traits, several limitations inherent to the study design should be acknowledged.

As this research is based on field sampling across natural populations rather than controlled experimental manipulation, causal inference among climate, soil properties, and host genetic effects remains inherently limited. In particular, the absence of detailed soil physicochemical measurements (e.g., pH, organic matter, nutrient availability, texture, and moisture) constrains our ability to fully disentangle the relative contributions of soil characteristics and climate to the observed microbial and phenotypic variation. Soil properties are well-established primary determinants of rhizosphere community composition and plant performance, and their covariation with climatic gradients may confound the interpretation of environmental drivers in natural settings. Therefore, the marker taxa identified in this study should be interpreted as indicators of site-associated community structure and host population context rather than definitive functional drivers of phenotypic traits. The associations reported here therefore reflect pattern-based relationships rather than direct causal mechanisms.

In addition, genetic differentiation among host populations may independently influence rhizosphere microbial assembly through genotype-specific root exudation patterns, immune regulation, and nutrient acquisition strategies. Thus, both abiotic filtering and host genetic background may jointly shape the microbial patterns observed across sites.

A further methodological consideration concerns the climatic data employed in this study. The climatic variables were derived from publicly available gridded datasets (WorldClim), and therefore may not precisely reflect the actual microclimatic conditions at each specific sampling location. Such discrepancies between gridded estimates and site-level climate may introduce some uncertainty into the observed associations among climate, rhizosphere microbial communities, and plant phenotypic variation.

Future studies incorporating site-specific climatic monitoring, comprehensive soil physicochemical characterization, common garden or reciprocal transplant experiments, and genomic analyses of host populations will be essential to disentangle environmental filtering from host genetic effects and to refine estimates of climate–microbiome–phenotype relationships. Such integrative approaches will provide stronger mechanistic insight into how environmental heterogeneity and host evolutionary divergence jointly shape rhizosphere community assembly and phenotypic differentiation in wild populations.

## 5 Conclusion

In summary, this study indicates that environmental heterogeneity is associated with significant phenotypic variation among *R. roxburghii* populations, particularly in reproductive traits such as fruit size, and that different populations exhibit distinct growth–reproduction patterns. Integrative analyses of rhizosphere microbial communities revealed consistent associations between fungal taxa and key phenotypic traits, with fungi showing broader correlations with fruit length and width compared with other variable sets. Climatic variables were also associated with variation in stem diameter, and shared variation between climate and rhizosphere microbes was observed, highlighting the statistical covariation of abiotic and biotic factors in relation to plant traits. However, the absence of soil physicochemical data and genomic characterization of host populations, together with the use of gridded climatic datasets, limits causal interpretation and precludes rigorous evaluation of the relative contributions of microbes and climate. The microbial taxa identified here should therefore be interpreted as indicators of community patterns associated with environmental heterogeneity and host population context rather than definitive functional drivers.

Overall, these findings emphasize the ecological relevance of rhizosphere microbial communities—particularly fungi—in heterogeneous environments at a pattern-based level. The results provide a regional-scale framework for understanding environment–microbiome–phenotype associations in *R. roxburghii* and offer a foundation for future studies integrating soil characterization, precise climatic monitoring, and host genomic data to further clarify underlying mechanisms.

## Data Availability

The original contributions presented in the study are publicly available. This data can be found at: https://doi.org/10.5281/zenodo.18754231. The data are now publicly accessible in an open repository.
