# Supplementary material for: Rhizosphere microbial patterns and climatic correlates of phenotypic variation in Rosa roxburghii
Source: Front Microbiol. 2026 Mar 31;17:1762588. doi: 10.3389/fmicb.2026.1762588 (PMC13076311; doi:10.3389/fmicb.2026.1762588)
Supplement: Supplementary file 1 [file Table_1.docx]

Supplementary Material

# Supplementary Figures and Tables

## Supplementary Tables

Supplementary Table S1. The latitude–longitude and elevation information of the different *Rosa roxburghii* populations’ sampling sites.

| **Sample** | **Longitude** | **Latitude** | **Elevation** | **Group** |
| --- | --- | --- | --- | --- |
| DS1 | 107.7099 | 25.7626 | 789.22 | DS |
| DS2 | 107.7017 | 25.7626 | 775.64 | DS |
| DS3 | 107.7078 | 25.7616 | 794.61 | DS |
| DS4 | 107.7075 | 25.7615 | 801.42 | DS |
| DS5 | 107.7073 | 25.7614 | 804.18 | DS |
| RH1 | 106.5392 | 27.9702 | 1010.5 | RH |
| RH2 | 106.539 | 27.9702 | 1049.8 | RH |
| RH3 | 106.5409 | 27.9708 | 982.4 | RH |
| RH4 | 106.5372 | 27.972 | 1020.4 | RH |
| RH5 | 106.5362 | 27.9726 | 994.4 | RH |
| LPS1 | 105.3737 | 26.3084 | 1299.52 | LPS |
| LPS2 | 105.3738 | 26.3084 | 1301.83 | LPS |
| LPS3 | 105.3732 | 26.308 | 1297.22 | LPS |
| LPS4 | 105.3753 | 26.314 | 1327.9 | LPS |
| LPS5 | 105.3755 | 26.3138 | 1326.64 | LPS |
| LL1 | 106.9777 | 26.4266 | 1360.36 | LL |
| LL2 | 106.9778 | 26.4267 | 1367.62 | LL |
| LL3 | 106.9776 | 26.4267 | 1369.52 | LL |
| LL4 | 106.9776 | 26.4266 | 1365.9 | LL |
| LL5 | 106.9775 | 26.4266 | 1357.97 | LL |
| SQ1 | 108.2168 | 27.6245 | 648.77 | SQ |
| SQ2 | 108.2167 | 27.6244 | 648.89 | SQ |
| SQ3 | 108.2165 | 27.6243 | 653.11 | SQ |
| SQ4 | 108.2169 | 27.6245 | 652.31 | SQ |
| SQ5 | 108.2168 | 27.6246 | 656.78 | SQ |

Supplementary Table S2. Phenotypic data of Rosa roxburghii populations in heterogeneous environments

| **Sample** | **Plant height** | **Crown width** | **Stem diameter** | **Leaf length** | **Leaf width** | **Fruit length** | **Fruit width** | **Fresh weight** | **Group** |
| --- | --- | --- | --- | --- | --- | --- | --- | --- | --- |
| DS1 | 130 | 185 | 3 | 2.22 | 0.96 | 2.62 | 3.14 | 7.8 | DS |
| DS2 | 90 | 177.5 | 1.7 | 2.38 | 1.5 | 2.26 | 2.78 | 5.764 | DS |
| DS3 | 120 | 135 | 2.5 | 2.24 | 1 | 2.02 | 2.6 | 5.268 | DS |
| DS4 | 150 | 100 | 3.2 | 1.98 | 0.74 | 2.2 | 2.82 | 6.026 | DS |
| DS5 | 160 | 210 | 4 | 2.06 | 0.82 | 2.16 | 2.78 | 5.942 | DS |
| RH1 | 121 | 112 | 1.6 | 2.56 | 0.5 | 2.04 | 2.6 | 6.172 | RH |
| RH2 | 119 | 133.5 | 1.7 | 2.3 | 1.22 | 2.16 | 2.76 | 7.848 | RH |
| RH3 | 153 | 150.5 | 2.1 | 2.44 | 1.3 | 2.42 | 2.62 | 5.138 | RH |
| RH4 | 123 | 93.5 | 2 | 2.1 | 1.24 | 2.1 | 2.42 | 4.474 | RH |
| RH5 | 141 | 136 | 2.1 | 2.16 | 2 | 2.2 | 2.66 | 5.158 | RH |
| LPS1 | 180 | 138.5 | 3.5 | 1.82 | 1.18 | 2.1 | 2.66 | 4.816 | LPS |
| LPS2 | 185 | 165 | 4 | 2.42 | 1.86 | 2.36 | 2.78 | 5.578 | LPS |
| LPS3 | 146 | 153.5 | 5.5 | 1.5 | 0.78 | 2.06 | 2.26 | 3.624 | LPS |
| LPS4 | 90 | 116 | 2.2 | 2.4 | 1.4 | 2.18 | 2.76 | 5.106 | LPS |
| LPS5 | 125 | 151 | 3.2 | 1.98 | 2.98 | 3.98 | 4.98 | 5.98 | LPS |
| LL1 | 122 | 129 | 4.2 | 2.02 | 1.26 | 2.32 | 2.98 | 6.054 | LL |
| LL2 | 160 | 122.5 | 2.8 | 1.91 | 2.91 | 3.91 | 4.91 | 5.91 | LL |
| LL3 | 130 | 137.5 | 3 | 1.94 | 2.94 | 3.94 | 4.94 | 5.94 | LL |
| LL4 | 130 | 145 | 4 | 2.27 | 3.27 | 4.27 | 5.27 | 6.27 | LL |
| LL5 | 100 | 165 | 3.5 | 1.9 | 1.1 | 2.52 | 3.2 | 7.986 | LL |
| SQ1 | 90 | 95 | 1.47 | 2.52 | 1.54 | 2.7 | 3.18 | 9.132 | SQ |
| SQ2 | 96 | 102.5 | 1.7 | 2.24 | 1.18 | 2.24 | 2.72 | 5.884 | SQ |
| SQ3 | 110 | 147.5 | 1.7 | 2.88 | 1.62 | 2.72 | 3.08 | 8.032 | SQ |
| SQ4 | 149 | 143.5 | 1.2 | 2.94 | 1.74 | 2.26 | 2.76 | 5.89 | SQ |
| SQ5 | 87 | 91 | 1.1 | 2.19 | 3.19 | 4.19 | 5.19 | 6.19 | SQ |

Supplementary Table S3. Climatic information of *Rosa roxburghii* populations from different locations obtained based on Wordclim

| **Sample** | **bio_1** | **bio_2** | **bio_3** | **bio_4** | **bio_5** | **bio_6** | **bio_7** |
| --- | --- | --- | --- | --- | --- | --- | --- |
| DS1 | 16.5 | 7.8 | 29.0 | 708.8 | 29.7 | 2.9 | 26.8 |
| DS2 | 16.5 | 7.7 | 29.0 | 706.7 | 29.6 | 2.9 | 26.7 |
| DS3 | 16.5 | 7.7 | 29.0 | 706.7 | 29.6 | 2.9 | 26.7 |
| DS4 | 16.5 | 7.7 | 29.0 | 706.7 | 29.6 | 2.9 | 26.7 |
| DS5 | 16.5 | 7.7 | 29.0 | 706.7 | 29.6 | 2.9 | 26.7 |
| RH1 | 15.5 | 7.2 | 26.4 | 730.6 | 30.0 | 2.5 | 27.5 |
| RH2 | 15.5 | 7.2 | 26.4 | 730.6 | 30.0 | 2.5 | 27.5 |
| RH3 | 15.5 | 7.2 | 26.4 | 730.6 | 30.0 | 2.5 | 27.5 |
| RH4 | 15.5 | 7.2 | 26.4 | 730.6 | 30.0 | 2.5 | 27.5 |
| RH5 | 15.5 | 7.2 | 26.4 | 730.6 | 30.0 | 2.5 | 27.5 |
| LPS1 | 15.0 | 7.9 | 31.8 | 622.8 | 26.9 | 2.1 | 24.8 |
| LPS2 | 15.0 | 7.9 | 31.8 | 622.8 | 26.9 | 2.1 | 24.8 |
| LPS3 | 15.0 | 7.9 | 31.8 | 622.8 | 26.9 | 2.1 | 24.8 |
| LPS4 | 15.6 | 7.9 | 31.9 | 630.4 | 27.5 | 2.6 | 24.9 |
| LPS5 | 15.6 | 7.9 | 31.9 | 630.4 | 27.5 | 2.6 | 24.9 |
| LL1 | 14.6 | 7.5 | 28.5 | 691.5 | 27.4 | 1.2 | 26.2 |
| LL2 | 14.6 | 7.5 | 28.5 | 691.5 | 27.4 | 1.2 | 26.2 |
| LL3 | 14.6 | 7.5 | 28.5 | 691.5 | 27.4 | 1.2 | 26.2 |
| LL4 | 14.6 | 7.5 | 28.5 | 691.5 | 27.4 | 1.2 | 26.2 |
| LL5 | 14.6 | 7.5 | 28.5 | 691.5 | 27.4 | 1.2 | 26.2 |
| SQ1 | 17.0 | 7.5 | 26.1 | 776.6 | 32.0 | 3.1 | 28.9 |
| SQ2 | 17.0 | 7.5 | 26.1 | 776.6 | 32.0 | 3.1 | 28.9 |
| SQ3 | 17.0 | 7.5 | 26.1 | 776.6 | 32.0 | 3.1 | 28.9 |
| SQ4 | 17.0 | 7.5 | 26.1 | 776.6 | 32.0 | 3.1 | 28.9 |
| SQ5 | 17.0 | 7.5 | 26.1 | 776.6 | 32.0 | 3.1 | 28.9 |
| **Sample** | **bio_8** | **bio_9** | **bio_10** | **bio_11** | **bio_12** | **bio_13** | **bio_14** |
| DS1 | 23.2 | 7.1 | 24.7 | 7.1 | 1265.8 | 254.4 | 29.2 |
| DS2 | 23.2 | 7.1 | 24.6 | 7.1 | 1258.4 | 252.8 | 29.3 |
| DS3 | 23.2 | 7.1 | 24.6 | 7.1 | 1258.4 | 252.8 | 29.3 |
| DS4 | 23.2 | 7.1 | 24.6 | 7.1 | 1258.4 | 252.8 | 29.3 |
| DS5 | 23.2 | 7.1 | 24.6 | 7.1 | 1258.4 | 252.8 | 29.3 |
| RH1 | 22.3 | 5.9 | 24.1 | 5.9 | 1102.3 | 209.7 | 15.3 |
| RH2 | 22.3 | 5.9 | 24.1 | 5.9 | 1102.3 | 209.7 | 15.3 |
| RH3 | 22.3 | 5.9 | 24.1 | 5.9 | 1102.3 | 209.7 | 15.3 |
| RH4 | 22.3 | 5.9 | 24.1 | 5.9 | 1102.3 | 209.7 | 15.3 |
| RH5 | 22.3 | 5.9 | 24.1 | 5.9 | 1102.3 | 209.7 | 15.3 |
| LPS1 | 20.9 | 6.5 | 22.0 | 6.5 | 1191.6 | 279.3 | 17.2 |
| LPS2 | 20.9 | 6.5 | 22.0 | 6.5 | 1191.6 | 279.3 | 17.2 |
| LPS3 | 20.9 | 6.5 | 22.0 | 6.5 | 1191.6 | 279.3 | 17.2 |
| LPS4 | 21.6 | 7.0 | 22.7 | 7.0 | 1186.5 | 279.4 | 17.3 |
| LPS5 | 21.6 | 7.0 | 22.7 | 7.0 | 1186.5 | 279.4 | 17.3 |
| LL1 | 21.2 | 5.4 | 22.6 | 5.4 | 1179.4 | 253.3 | 18.9 |
| LL2 | 21.2 | 5.4 | 22.6 | 5.4 | 1179.4 | 253.3 | 18.9 |
| LL3 | 21.2 | 5.4 | 22.6 | 5.4 | 1179.4 | 253.3 | 18.9 |
| LL4 | 21.2 | 5.4 | 22.6 | 5.4 | 1179.4 | 253.3 | 18.9 |
| LL5 | 21.2 | 5.4 | 22.6 | 5.4 | 1179.4 | 253.3 | 18.9 |
| SQ1 | 24.4 | 7.0 | 26.3 | 7.0 | 1163.4 | 210.5 | 23.0 |
| SQ2 | 24.4 | 7.0 | 26.3 | 7.0 | 1163.4 | 210.5 | 23.0 |
| SQ3 | 24.4 | 7.0 | 26.3 | 7.0 | 1163.4 | 210.5 | 23.0 |
| SQ4 | 24.4 | 7.0 | 26.3 | 7.0 | 1163.4 | 210.5 | 23.0 |
| SQ5 | 24.4 | 7.0 | 26.3 | 7.0 | 1163.4 | 210.5 | 23.0 |
| **Sample** | **bio_15** | **bio_16** | **bio_17** | **bio_18** | **bio_19** | **Group** |  |
| DS1 | 69.2 | 625.9 | 97.2 | 549.5 | 97.2 | DS |  |
| DS2 | 69.4 | 623.7 | 95.1 | 546.9 | 95.1 | DS |  |
| DS3 | 69.4 | 623.7 | 95.1 | 546.9 | 95.1 | DS |  |
| DS4 | 69.4 | 623.7 | 95.1 | 546.9 | 95.1 | DS |  |
| DS5 | 69.4 | 623.7 | 95.1 | 546.9 | 95.1 | DS |  |
| RH1 | 70.5 | 539.1 | 53.9 | 480.6 | 53.9 | RH |  |
| RH2 | 70.5 | 539.1 | 53.9 | 480.6 | 53.9 | RH |  |
| RH3 | 70.5 | 539.1 | 53.9 | 480.6 | 53.9 | RH |  |
| RH4 | 70.5 | 539.1 | 53.9 | 480.6 | 53.9 | RH |  |
| RH5 | 70.5 | 539.1 | 53.9 | 480.6 | 53.9 | RH |  |
| LPS1 | 82.9 | 616.3 | 59.7 | 608.2 | 59.7 | LPS |  |
| LPS2 | 82.9 | 616.3 | 59.7 | 608.2 | 59.7 | LPS |  |
| LPS3 | 82.9 | 616.3 | 59.7 | 608.2 | 59.7 | LPS |  |
| LPS4 | 82.9 | 616.6 | 59.9 | 604.7 | 59.9 | LPS |  |
| LPS5 | 82.9 | 616.6 | 59.9 | 604.7 | 59.9 | LPS |  |
| LL1 | 74.6 | 608.7 | 70.1 | 529.0 | 70.1 | LL |  |
| LL2 | 74.6 | 608.7 | 70.1 | 529.0 | 70.1 | LL |  |
| LL3 | 74.6 | 608.7 | 70.1 | 529.0 | 70.1 | LL |  |
| LL4 | 74.6 | 608.7 | 70.1 | 529.0 | 70.1 | LL |  |
| LL5 | 74.6 | 608.7 | 70.1 | 529.0 | 70.1 | LL |  |
| SQ1 | 63.8 | 543.8 | 82.5 | 450.1 | 82.5 | SQ |  |
| SQ2 | 63.8 | 543.8 | 82.5 | 450.1 | 82.5 | SQ |  |
| SQ3 | 63.8 | 543.8 | 82.5 | 450.1 | 82.5 | SQ |  |
| SQ4 | 63.8 | 543.8 | 82.5 | 450.1 | 82.5 | SQ |  |
| SQ5 | 63.8 | 543.8 | 82.5 | 450.1 | 82.5 | SQ |  |

Note: The definitions of the climatic factors are provided in Supplementary Table S4.

Supplementary Table S4. Definitions of the climatic factors

| **Bio Factors** | **Description** | **Unit** |
| --- | --- | --- |
| bio_1 | Annual Mean Temperature | °C |
| bio_2 | Mean Diurnal Range (Mean of monthly (max temp - min temp)) | °C |
| bio_3 | Isothermality (bio2/bio7) (×100) | - |
| bio_4 | Temperature Seasonality (standard deviation ×100) | °C |
| bio_5 | Max Temperature of Warmest Month | °C |
| bio_6 | Min Temperature of Coldest Month | °C |
| bio_7 | Temperature Annual Range (bio5-bio6) | °C |
| bio_8 | Mean Temperature of Wettest Quarter | °C |
| bio_9 | Mean Temperature of Driest Quarter | °C |
| bio_10 | Mean Temperature of Warmest Quarter | °C |
| bio_11 | Mean Temperature of Coldest Quarter | °C |
| bio_12 | Annual Precipitation | mm |
| bio_13 | Precipitation of Wettest Month | mm |
| bio_14 | Precipitation of Driest Month | mm |
| bio_15 | Precipitation Seasonality (Coefficient of Variation) | - |
| bio_16 | Precipitation of Wettest Quarter | mm |
| bio_17 | Precipitation of Driest Quarter | mm |
| bio_18 | Precipitation of Warmest Quarter | mm |
| bio_19 | Precipitation of Coldest Quarter | mm |

## Supplementary Figures


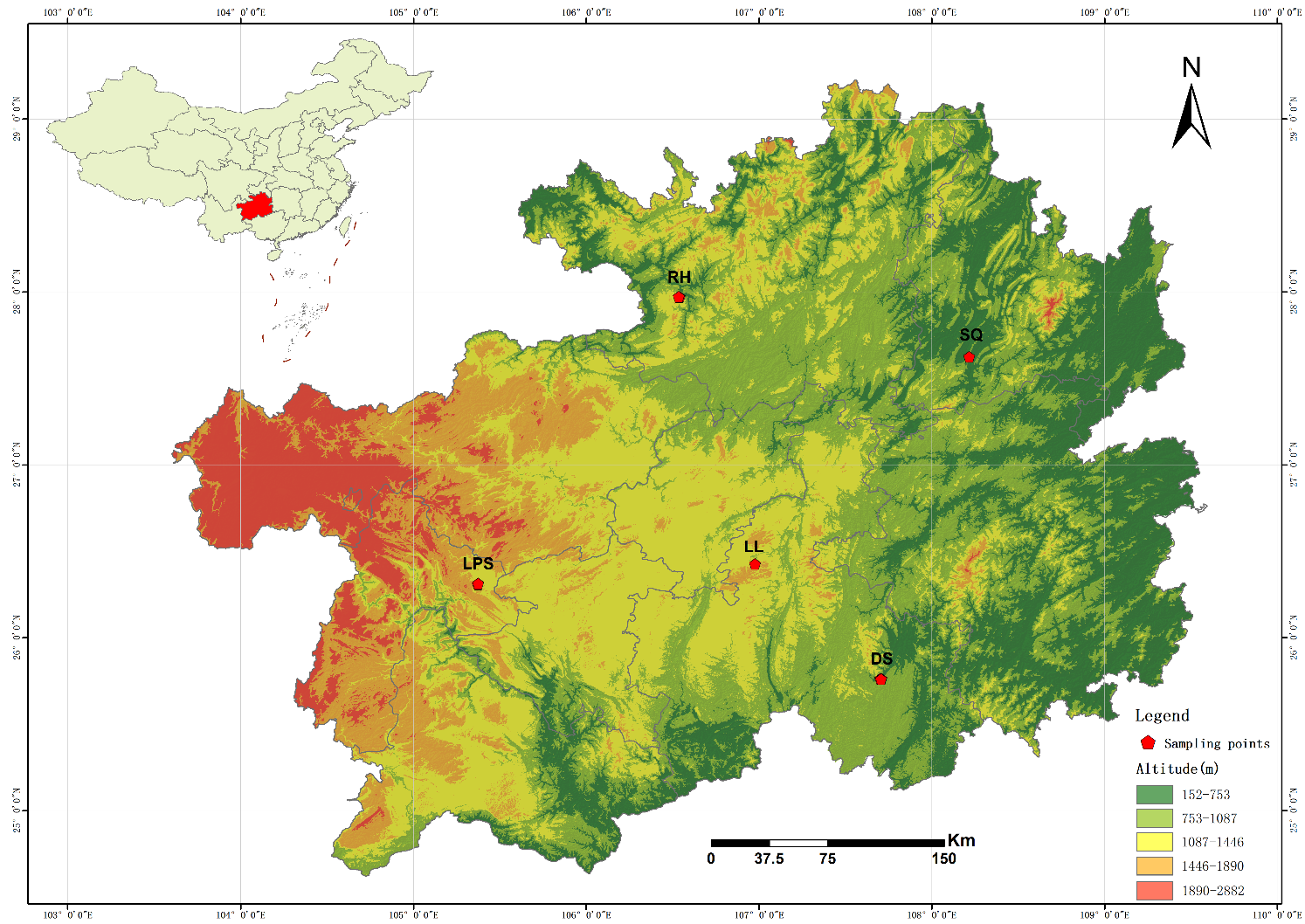


**Figure S1.** Geographic distribution of the five *Rosa roxburghii* sampling sites. The horizontal and vertical axes represent longitude and latitude, respectively. Different colors indicate the altitudinal gradient, and red pentagons denote the specific sampling locations, with five individuals collected per site. Map data were obtained from the National Geospatial Information Public Service Platform of China (Approval number: GS(2019)1822).


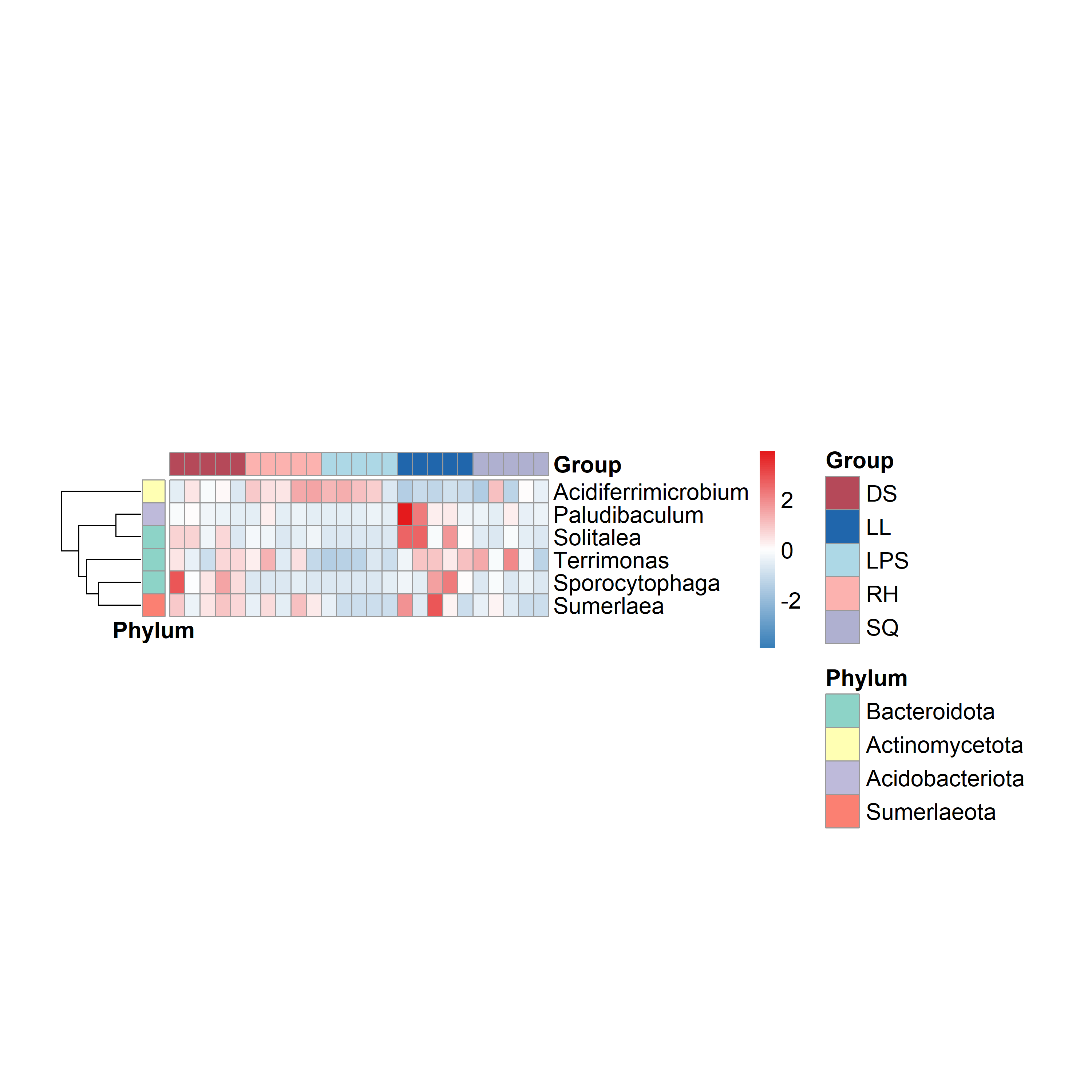


**Figure S2.** Heatmap of the abundances of key rhizosphere bacterial genera selected by multiple models. The x-axis represents different *Rosa roxburghii* samples and their groups, while the y-axis represents the bacterial genera and their corresponding phyla. Higher abundances are shown in red, whereas lower abundances are shown in blue.


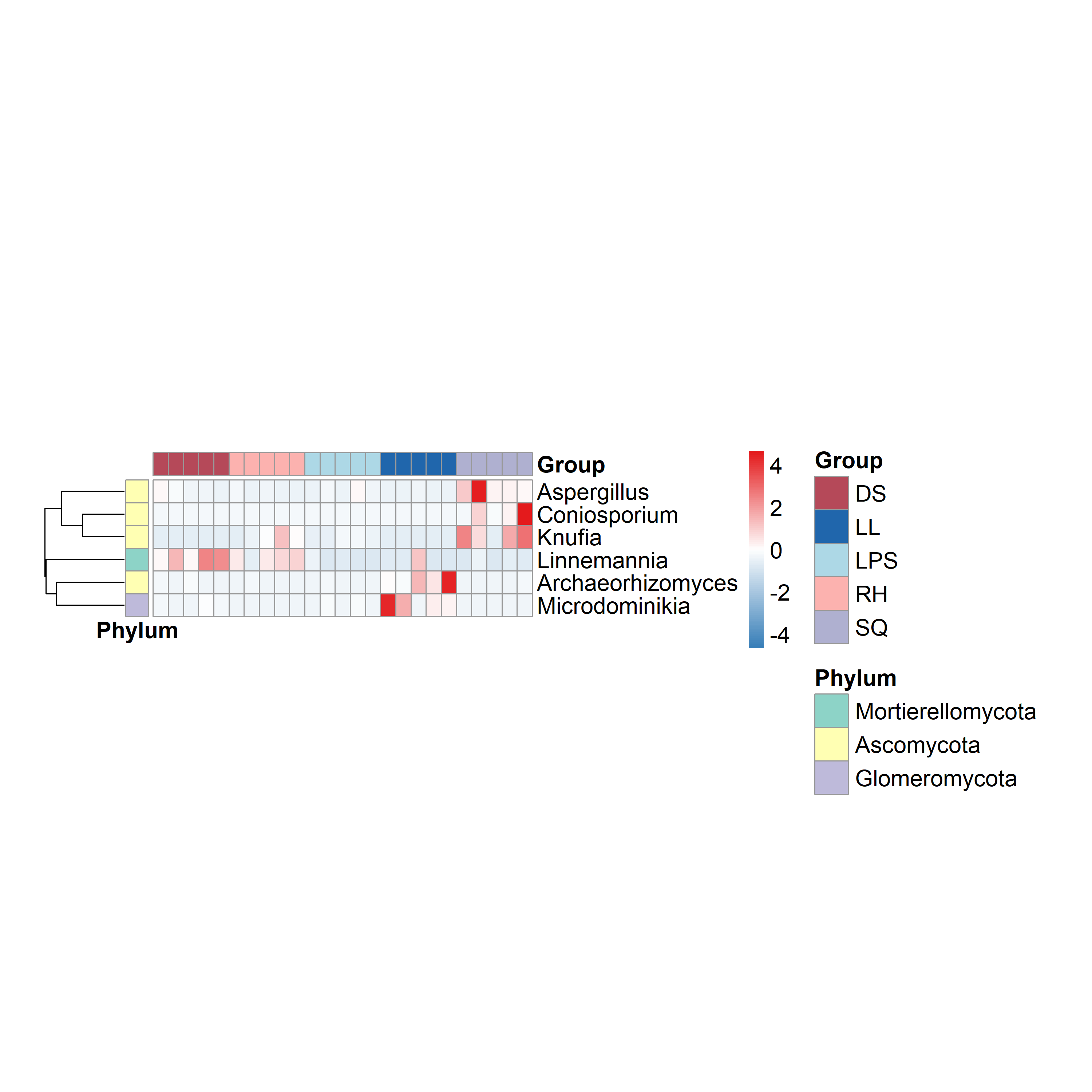


**Figure S3.** Heatmap of the abundances of key rhizosphere fungal genera selected by multiple models. The x-axis represents different *Rosa roxburghii* samples and their groups, while the y-axis represents the fungal genera and their corresponding phyla. Higher abundances are shown in red, whereas lower abundances are shown in blue.
